# Prioritization and Evaluation of Flooding Tolerance Genes in Soybean [*Glycine max* (L.) Merr.]

**DOI:** 10.3389/fgene.2020.612131

**Published:** 2021-01-27

**Authors:** Mu-Chien Lai, Zheng-Yuan Lai, Li-Hsin Jhan, Ya-Syuan Lai, Chung-Feng Kao

**Affiliations:** ^1^Department of Agronomy, College of Agriculture and Natural Resources, National Chung Hsing University, Taichung, Taiwan; ^2^Advanced Plant Biotechnology Center, National Chung Hsing University, Taichung, Taiwan

**Keywords:** soybean, flooding tolerance, genomic data meta-analysis, gene prioritization, gene-set enrichment analysis

## Abstract

Soybean [*Glycine max* (L.) Merr.] is one of the most important legume crops abundant in edible protein and oil in the world. In recent years there has been increasingly more drastic weather caused by climate change, with flooding, drought, and unevenly distributed rainfall gradually increasing in terms of the frequency and intensity worldwide. Severe flooding has caused extensive losses to soybean production and there is an urgent need to breed strong soybean seeds with high flooding tolerance. The present study demonstrates bioinformatics big data mining and integration, meta-analysis, gene mapping, gene prioritization, and systems biology for identifying prioritized genes of flooding tolerance in soybean. A total of 83 flooding tolerance genes (FTgenes), according to the appropriate cut-off point, were prioritized from 36,705 test genes collected from multidimensional genomic features linking to soybean flooding tolerance. Several validation results using independent samples from SoyNet, genome-wide association study, SoyBase, GO database, and transcriptome databases all exhibited excellent agreement, suggesting these 83 FTgenes were significantly superior to others. These results provide valuable information and contribution to research on the varieties selection of soybean.

## Introduction

Soybean [*Glycine max* (L.) Merr] is an important food crop worldwide that provides an essential source of anthocyanins and isoflavones for human beings (Cederroth and Nef, 2009). Previous studies have shown that soybean isoflavones can decelerate at the apoptotic rate of the cerebral cortex of rats and minimize the occurrence of ischemic stroke (Schreihofer et al., 2005; Burguete et al., 2006; Lovekamp-Swan et al., 2007). Some anthocyanin compounds possess novel antioxidant capacity and have neuroprotective effects on the central nervous system (Wang et al., 2010; Lu et al., 2012) that are beneficial for conditions such as Alzheimer’s disease and Parkinson’s disease (Huang et al., 2009; Zhang et al., 2019). Therefore, maintaining a stable supply of soybean is important for the treatment of complex diseases.

Even though global soybean production has steadily risen in recent years (FAOstat, faostat.fao.org), there is still a shortage of food supply for human beings due to increases in natural disasters. Soybean is stress-sensitive and is particularly affected by flooding (Hou and Thseng, 1991; VanToai et al., 2010), one of the major abiotic stresses that can cause enormous losses in soybean production (Rosenzweig et al., 2002; Ahmed et al., 2013). In 2011, flooding and drought stress accounted for more than 70% of the reduction of major crops in the United States (Bailey-Serres et al., 2012). According to the degree of damage, flooding stress is classified into waterlogging and submergence, which means the water covers only the root system and the water covers both the shoot and the root system, respectively (Fukao et al., 2019). The roots and shoots of soybeans grow much more slowly if they are submerged in water (Oosterhuis et al., 1990). The nitrogen fixation in their root systems is also impeded (Board, 2008; Youn et al., 2008). As the time of submergence increases, it interferes with photosynthesis, stomatal conductance, and the absorption of nutrients (Jackson et al., 2009). Therefore, breeding work for flooding-tolerant soybean varieties is imperative.

Many studies have examined the selection of flood-tolerant soybean varieties. Shannon et al. (2005) conducted flood-tolerance experiments using 350 soybean germplasm lines and found that six lines (Archer, Misuzudaiz, PI408105A, PI561271, PI567651, and PI567343) were highly related to flooding tolerance during early reproductive stages. Wu et al. (2017) evaluated 722 soybean germplasms through foliar damage score and plant survival rate for accessing flooding tolerance during 2012 and 2016. Eleven flooding-tolerant lines (PI408105A, PI471931, PI471938, RA-452, Walters, R11-6870, R10-4892, R10-230, R07-6669, R07-2001, and R04-342) were identified which showed consistent flooding tolerance during 4- to 5- year continual evaluations. Meanwhile, the genetic information of several of these were involved with flood-tolerant related studies in soybean had been reported and accumulated. For instance, in gene expression studies, Chen et al. (2016) used RNA-Seq transcriptome profiling to identify differentially expressed genes and found that 3,498 of them were significantly associated with flooding tolerance. Since flooding tolerance was a quantitative trait, many previous studies used linkage mapping analysis to find quantitative trait locus (QTLs) (VanToai et al., 2001; Sayama et al., 2009; Nguyen et al., 2012, 2017). Cornelious et al. (2005) used 912 simple sequence repeat (SSR) markers to select QTLs and identified 17 SSR markers that were significantly associated with flooding tolerance in soybean. In the pathway function regulation platform, a total of 31 genes were identified under flooding stress by using quantitative reverse transcription-polymerase chain reaction (qRT-PCR). These genes were linked to the pathways including protein synthesis, nucleotide metabolism, hormone metabolism and glycolysis signaling which were induced by submergence conditions (Yin et al., 2017). Although previous studies were abundant, it was still costly and time-consuming for experimental validation of each of the flooding tolerance candidate genes (Rhee and Mutwil, 2014; Zhai et al., 2016). Furthermore, the data collected from previous studies had large batch effects (Goh et al., 2017), thus making it thorny to unify and integrate. Consequently, a gene prioritization technique, based on the computational method was developed.

The first research applying gene prioritization to plants was published by Xia et al. (2013). They utilized a keyword search in the NCBI PubMed database to collect data related to rice blight and used chaos-algorithm based classifiers to identify 74 blight resistance-related candidate genes in rice. Recently, network analysis advanced the development of gene prioritization. Zhai et al. (2016) proposed rank aggregation-based data fusion for a gene prioritization (RAP) method that integrated RafSee and AraNet v2 prioritization algorithms, a total of 380 flowering-time genes of *Arabidopsis thaliana* were identified. They found that the prioritized genes identified by the RAP method had a higher ranking in comparison to those that identified by AraNet v2. However, the limitation of the RAP gene prioritization method is that the prediction ability decreases when the number of core genes is deficient. This indicates that the RAP gene prioritization method is not suitable for other crops with fewer known functional genes. In soybean, 59 prioritized genes were identified by using the SoyNet database, which contains 40,812 soybean genes and 1,940,284 links from several data platforms covering 72.8% of the soybean genome (Kim et al., 2017). Nevertheless, the prioritization of flooding tolerance genes (FTgenes) in soybean has not been elucidated to date.

In the present study, we mined and collected genetic information and databases from different data sources on flood-tolerant in soybean. Here we defined flooding tolerance genes (denoted as FTgenes) to be significantly associated with traits that are related to flooding tolerance or responded to the stress after flooding treatment was given during germination and vegetative stages of soybean. The flooding treatment mainly focused on submergence. Genes that regulated physiological mechanisms involving both submergence and waterlogging were also considered to extend the pool of genomic data collected from multiple dimensional data platforms.

To minimize possible selection bias and noise, we considered nine data platforms, including association mapping study (including genome-wide association study, GWAS), linkage mapping analysis, gene expression, pathway regulation, protein-protein interaction networks (PPIN), network analysis, proteomes, and text mining, as well as functional genomic data from model plants. A scoring and weighting scheme was proposed to extract, integrate, and prioritize genomic data across multidimensional data sources using meta-analysis and prioritization system. To avoid false positive results, all positive and negative results were considered and integrated to give a real value for every genetic locus to construct an evidence-based gene pool (denoted as test genes). We computed a weighted combined score summarized from multiple data sources for each of the test genes. Similarly, a set of core genes were established for prioritizing the test genes. A clear separation between the test genes and the core genes was determined to identify prioritized FTgenes. This provides a valuable contribution for subsequent research and soybean variety selection.

## Materials and Methods

**FIGURE 1:**
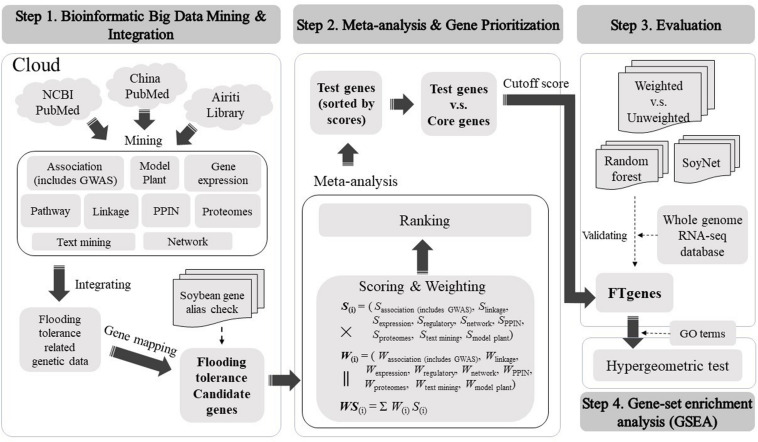
The framework of the present study.

We constructed a comprehensive framework to computationally optimize the test genes to construct prioritized FTgenes in soybean. There were four stages in the framework, including bioinformatic big data mining and integration, meta-analysis and gene prioritization, evaluation, and gene-set enrichment analysis. Detailed methods and materials used in this study were illustrated below (Figure 1).

### Test Genes and Core Genes

The test genes and the core genes were generated at the bioinformatics big data mining and integration stage. The collection of the test genes used text/data mining from multiple dimensional data sources. For detailed methods and approaches please refer to our proposed techniques described below in the “Bioinformatics Big Data Mining and Integration” sub-section. The establishment of the core genes was to provide an important basis for gene prioritization. The selection of the core genes was based on three criteria as follows. (1) Only genes that were reported in previous studies that significantly related to flooding tolerance or responded to flooding stress. These genes were also ranked in the top 2% of gene prioritization procedures. (2) Only genes that were significantly reported to be associated with flooding tolerance in network data platform. (3) Only genes that were significantly reported on at least four data platforms, where they scored higher than 40 in a weighted score and scored higher than 4 in each of the data platforms. We used the core genes to prioritize test genes according to their magnitude of association or response change to the stress from multiple data sources to search for prioritized FTgenes.

### Validation Databases

We used the whole genome expression database (RNA-seq data) of soybean seedling submergence published by Lin et al. (2019) as our validation databases. They investigated and recorded the expression change of soybean roots at four different time-points (3, 6, 12, and 24 h) after given submergence treatments. A total of 14,772, 17,017, 19,060, and 18,889 gene expression data were contained in the four time-point databases, respectively.

### Bioinformatics Big Data Mining and Integration

We collected genomic data that related to flooding tolerance or responded to the stress in soybean from the National Center for Biotechnology Information (NCBI) PubMed^1^, Airiti Library^2^, and China PubMed^3^. A keyword search method was applied to mine genomic data extracted from published journal articles and available open databases that were published before March 2020 that were relevant to our target. The keywords consist of combinations of three terms (crop, trait, and data platform). The keywords for “crop” terms were “soybean,” “cultured soybean,” and “glycine max.” The keywords for “trait” terms were “flooding tolerance,” “flooding stress,” “waterlogging,” and “submergence.” The keywords for “data platform” were “GWAS,” “association mapping,” “linkage mapping,” “gene expression,” “pathway regulation,” “PPIN,” “networks,” “proteomes,” and “text mining.” All genomic data, including journal articles and databases, collected from the cloud were examined and integrated carefully by experienced experts who are well-trained in big data mining for data management and data quality control. Studies that involved human trails, animal experiments, genetically modified organism studies, non-soybean studies, not applicable, and others that were irrelevant to the present study were excluded from big data integration.

The selection criteria of genomic data are illustrated below. In the association mapping data platform (including GWAS), only SNP markers having minor allele frequency (MAF) >5%, heterozygous allele calls <10%, call rate >90%, and *P*-value <0.05 were included. In the gene expression data platform, only genes with a *P*-value <0.05 and/or fold change (FC) >1.5 were considered. In linkage mapping and proteomes data platforms, only genetic data that *P*-value < 0.05 and/or logarithm of odds (LOD) >3 were mined. In the networks data platform, only genes that *P*-value <0.05 were collected. In the pathway regulation data platform, only pathways that were significantly reported to be associated with flooding tolerance were considered and we then screened out the genes involved in the pathways. In text mining data platform, we searched the keyword combinations of “gene symbol + crop + trait” using both MySQL and R package for parallel web crawling in the NCBI PubMed, Airiti Library, and China PubMed. The gene symbols were downloaded from SoyBase^4^. A hit was made if the journal article matched the keyword combinations. In the PPIN data platform, a total of 717,676 protein-protein interactions (regulations) were downloaded from the PlantRegMap^5^ in February 2020. We considered both positive findings and negative results to minimize possible false positive results and false negative results so that a precise evidence-based score for each genomic data could be calculated with fewer biases and noise.

To extend our method to summarize the roles of selected genes in regulating tolerance by integrating available genes from model plants, we included homologous genes from *A. thaliana* and *Medicago truncatula*. For the homologous gene data platform, protein sequences from the *Glycine max* genome were, respectively, aligned to those from the two model plants using BLASTP (Camacho et al., 2009). All protein sequence data, integrated from several studies (Schmutz et al., 2010; Tang et al., 2014; Cheng et al., 2017), were downloaded from the phytozome database^6^ (Goodstein et al., 2012). We searched for sequences with the highest similarity from alignment results to identify homologous proteins or genes corresponding to soybean genes. These homologous genes were further confirmed whether they were significantly reported as flooding related genes in *A. thaliana* and *M. truncatula*.

The genomic data collected in the present study included SNPs, genes, SSRs, and QTLs. We conducted gene mapping to assign all genomic data to a gene region using a window spanning 20 kb upstream to 20 kb downstream of the gene (Ravelombola et al., 2019). The principle of gene mapping was shown in Supplementary Figure 1. We notice that regions of QTL with longer than 5 centiMorgan (cM) in length were excluded (Liu et al., 2019) from big data integration to reduce possible false positive results and noise, in particular, QTLs across two different chromosomes. All genomic data were mapped into gene-level data corresponding to the correct gene version that was used in the original studies. Finally, we conducted gene version correspondence analysis to unambiguously match the above mapped gene-level data using the SoyBase Gene Model Correspondence Lookup tool^7^ to unify various gene versions (Glyma v1.0, Glyma v1.1, and Glyma v2.0) into Glyma v2.0 gene version. As a result, a set of test genes in the Glyma v2.0 version were established.

### Meta-Analysis and Gene Prioritization

We developed a scoring scheme according to the corresponding magnitude of association of flooding tolerance or response to flooding stress in soybean to address the issue of different types of data sources. The evidence from multidimensional data sources was in a wide range of different types of values, including association *P*-value, LOD, FC, score, degree, cluster coefficient, and correlation (*r*). The scoring systems for different platforms are different. Generally, we applied −*log*_10_⁡(*P*−value), |FC| and ⌊LOD⌋ to separately convert *P*-value, FC, and LOD into a score bounded within [0, 10]. The symbol of log(⋅), |⋅|, and ⌊⋅⌋ represents 10-based logarithm, absolute value, and floor function, respectively. We denoted *S*^M^ as a scoring system computed by using a transformation function described above, where the superscript letter *M* is a measurement characterized by a *P*-value, FC, LOD, degree, and *r*. In the association mapping data platform, we applied ^*SP*^ = −*log*_10_⁡(*P*−value) for scoring. In the linkage mapping and proteomes data platforms, we calculated max{^*SP*^,*S*^*FC*^,*S*^*degree*^}, and max{^*SP*^,*S*^*FC*^,*S*^*degree*^} separately for loci, respectively. In gene expression data platform, the max{^*SP*^,*S*^*FC*^} was applied for scoring. In the networks data platform, we quantified max{^*SP*^,*S*^*FC*^,^*Sr*^} to obtain a score range [1,6]. In the pathway regulation data platform, we proposed a reported frequency-based algorithm for the scoring of selected genes. In text mining and model plant data platforms, we scored 1 if the keyword search was made or the homologous gene was confirmed and scored 0 otherwise. In the PPIN data platform, we scored the regulation networks according to the strength of degree and cluster coefficient. If two or more types of different data were present for a gene within the same data platform, we computed a score for each data type and selected the maximal value as the score for the gene. A detailed scoring system can be found in Supplementary Table 1.

In the stage of weighting, we evaluated each of the selected genomic data using an impact factor corresponding to their published journal articles to access data reliability (Supplementary Table 2). For each gene, we calculated a weighted score using score multiply weight across multiple data platforms to quantify the importance of gene linking to flooding tolerance in soybean. Generally, the distribution of weighted scores for the test genes and the core genes are skewed to the right and skewed to the left, respectively. Hence, an optimal cutoff-score can be found to distinctly separate the two distributions of the test genes and the core genes. Only genes that scored greater than the cutoff-score were selected as prioritized FTgenes.

### Evaluation

Two approaches were applied to access the reliability and the robustness of the prioritized FTgenes using the RNA-seq data (Lin et al., 2019). First, we compared the prioritized FTgenes using a weighted scheme to examine whether the prioritized FTgenes showed a higher change to obtain smaller *P*-values (or larger FCs) than those using the unweighted scheme. Comparisons of the intersection, the difference of unweighted FTgenes from weighted FTgenes (denoted as FTgenes^*w\nw*^), and the difference of weighted FTgenes from unweighted FTgenes (denoted as FTgenes^*nw\w*^) were also examined.

We also compared the prioritized FTgenes (using weighted scheme) selected by our proposed algorithm with other existing methods, e.g., random forest algorithm (Rafsee) (Zhai et al., 2016) and network-based gene prioritization method (SoyNet) (Kim et al., 2017). Due to the difference of sources and characteristics for data mining between other methods and our study, we modified the random forest algorithm used in Rafsee without changing the idea and concept and applied it to our test genes. We modified the random forest algorithm as follows. (1) Bootstrap: we generated a subset of the same size from the test genes by sampling with replacement, and selected the top 83 genes with the highest weighted score (denoted as bootstrap genes). (2) Permutation: we randomly shuffled (i.e., under the null) the order of genes to break inherent structure of dependence between genes and the scores in each bootstrap subset, and selected the top 83 genes with highest weighted score (denoted as permutation genes). (3) Selection: we selected the bootstrap genes if the weighted score of the top 83 bootstrap gene was greater than the highest weighted score in permutation genes, and discarded them otherwise. (4) Loop: we repeated steps 1–3 until 10,000 sets of bootstrap genes were achieved. (5) Ranking: we counted gene frequencies for each set of 10,000 bootstrap genes, and selected the top 83 bootstrap genes with the highest frequencies as prioritized genes. The second method compared to our algorithm was SoyNet (Kim et al., 2017), which is a co-functional network webserver^8^. The SoyNet contained 40,812 soybean genes and 1,940,284 links collected from 21 distinct data types, covering 72.8% of the soybean genome. We conducted gene prioritization based on Bayesian statistics using the top 2,000 weighted genes using function II “Find context associated genes.” We selected the top 83 prioritized genes from 502 significant genes (*P*-value < 1.0×10^–8^). We compared our prioritized FTgenes to those two top genes prioritized using other methods described above for validation using the Wilcoxon rank-sum test, and a *P*-value was calculated based on 100,000 bootstrap samples for each method.

### Gene-Set Enrichment Analysis

The GeneOntology (GO; http://geneontology.org/) database collected abundant terms that were related to gene functions in soybean. According to the gene products, these terms can be classified into three categories: biological process (7,332 terms), cellular component (2,761 terms), and molecular function (3,199 terms). We applied gene-set enrichment analysis to investigate significantly enriched potential physiological regulation pathways, using the GO database and the prioritized FTgenes. The hypergeometric test was conducted to identify significantly enriched pathways, and adjusted *P*-values were calculated using the Bonferroni correction method to avoid false positive results.

## Results

### Bioinformatics Big Data Mining and Integration

In bioinformatics, big data mining, 66 journal articles, and 4 databases were qualified. Through quality control, we removed 17 articles irrelevant to our study, there were 49 journal articles, and 4 databases in the end (Table 1). We found 47,227, 2,014, 59, 47,931, and 376 genes, respectively, from gene expression, pathway regulatory, networks, PPIN, and the proteomes data platform. 79 SNPs and 66 SSR markers were found from association mapping and linkage mapping data platforms, respectively. A total of 106,263 genotype data were included in this study. Two RNA-seq databases (46,938 genes) (Nanjo et al., 2011; Chen et al., 2016) and one GO pathway regulation database (2,014 genes in 14 pathways) were included. In the PPIN database, a total of 717,676 gene pairs were included (PlantRegMap: http://plantregmap.cbi.pku.edu.cn/) (Supplementary Table 3 and Supplementary Material 1).

**TABLE 1:** Summary results of big data mining and bioinformatics across different data platforms for flooding tolerance in soybean.

**Data platform**	**Data mining results**		**Selected data sources**		**No. of genetic data extracted^*a*^**
	**No. of articles**	**No. of databases**	**No. of articles**	**No. of databases**	
Association mapping (includes GWAS)	8	0	2	0	79
Linkage mapping	10	0	7	0	66
Gene expression	11	2	9	2	47,227
Pathway regulatory	21	1	15	1	2,014
Gene networks	1	0	1	0	59
PPIN	0	1	0	1	47,931
Proteomes	4	0	4	0	376
Homologous gene	11	0	11	0	8,511
Total	66	4	49	4	106,263

*Abbreviation: GWAS, genome-wide association study; PPIN, protein-protein interaction network.^*a*^Genetic data include SNPs, genes, SSRs, and QTLs.*

In the stage of bioinformatics big data integration, genetic data including SNPs, SSRs, and QTLs are required to perform gene mapping so that every loci was mapped onto the gene level. A total of 79 SNPs and 66 SSRs were mapped to 117 genes and 296 genes, separately in association mapping and linkage mapping data platform. For the homologous gene data platform, a total of 34,738 and 50,188 potential homologous genes, respectively, from *A. thaliana* and *M. truncatula* were identified using BLASTP. To confirm these genes whether associated with flooding, we collected flooding-related studies in two model plants to extract their candidate genes. In a total of 8,511, *A. thaliana* candidate genes were found in 11 papers. Unfortunately, we did not find any genetic information in *M. truncatula*. Next, the overlap between homologous and candidate genes was verified to compute homo.score for tested genes. Our results showed that 11,275 tested genes were reported in previous studies that related to flooding stress. In total, 36,705 test genes were constructed from multidimensional data platforms for meta-analysis and gene prioritization.

Table 2 shows the summary results of genomic data on flooding tolerance or response to flooding stress indices at a particular growth stage in soybean. In the germination stage, differentially expressed genes in root and/or leaf tissues were measured in gene expression, pathway regulators, and proteomes studies. During germination-reproductive stages, three indices (flooding injury scores, germination rate, and normal seeding rates) were often used to evaluate the degree of flooding tolerance in linkage and association mapping studies.

**TABLE 2:** Summary results of flooding tolerance index from each data platform.

**Platform**	**Growth stage**	**Flooding tolerance related traits**	**References**
Association (including GWAS)	Reproductive stage	Flooding injury scores, level 1–5	Ye et al. (2018)
Association (including GWAS)	Germination stage	Germination rate, normal seedling rate, electric conductivity	Yu et al. (2019)
Association (including GWAS)	Reproductive stage	Foliar damage score	Wu et al. (2019)
Gene expression	Germination stage	Collect root and hypocotyl and detect the expression of RNAs and proteins	Komatsu et al. (2009)
Gene expression	Germination stage	Extract RNA form roots (including hypocotyl)	Nanjo et al. (2011)
Gene expression	Germination stage	Extract RNA form roots (including hypocotyl)	Nishizawa et al. (2013)
Gene expression	Germination stage	Extract RNA form roots (including hypocotyl)	Song et al. (2018)
Gene expression	V1_Vegetative stage	Extract RNA from root tissues	Nakayama et al. (2014)
Gene expression	V1_Vegetative stage	qRT-PCR for roots	Valliyodan et al. (2014)
Gene expression	V4_Vegetative stage	Use leaves to do RNA-sequencing and real-time PCR analysis	Chen et al. (2016)
Gene expression	V5_Vegetative stage	Use leaves to do RNA-sequencing, RT-PCR, and qPCR	Syed et al. (2015)
Gene expression	Vegetative stage	Extract RNA from root tissues	Nakayama et al. (2017)
Linkage mapping	Germination stage	Germination rate, healthy growth rate, damage of roots and shoots	Rizal and Karki (2011)
Linkage mapping	Germination stage	100 seed weight, germination rate, normal seedling rate	Sayama et al. (2009)
Linkage mapping	R1_Reproductive stage	Plant height, seed yield	VanToai et al. (2001)
Linkage mapping	R1_Reproductive stage	Flooding tolerance score, score1–5	Nguyen et al. (2012)
Linkage mapping	R2_Reproductive stage	Plant injury score (score 1–9)	Cornelious et al. (2005)
Linkage mapping	V3_Vegetative stage	Flooding tolerance index (seed weight of treated plants divided by that of control plants), days to flowering, days to maturity, plant height, branch number, pod number, seed number, seed weight, 100-seed weight	Githiri et al. (2006)
Pathway	Germination stage	Hypocotyl	Khan et al. (2015)
Pathway	Germination stage	Roots	Khatoon et al. (2012)
Pathway	Germination stage	Roots	Komatsu et al. (2013)
Pathway	Germination stage	Roots	Mustafa and Komatsu (2014)
Pathway	Germination stage	Roots and cotyledons	Mustafa et al. (2015)
Pathway	Germination stage	Cotyledons	Nanjo et al. (2010)
Pathway	Germination stage	Roots including hypocotyl	Nanjo et al. (2011)
Pathway	Germination stage	5 mm long root tips	Nanjo et al. (2012)
Pathway	Germination stage	Root tips	Yin and Komatsu (2016)
Pathway	Germination stage	Root tips	Yin et al. (2014)
Pathway	Germination stage	Root tips	Yin and Komatsu (2015)
Pathway	Germination stage	Root tips	Yin et al. (2016)
Pathway	Germination stage	Root tips, including hypocotyl and cotyledons	Yin et al. (2017)
Proteomes	Germination stage	Plant survival rate, lacking appartent damage, lateral root development, radicle elongation rate	Nanjo et al. (2014)
Proteomes	Germination stage	Roots, hypocotyls, cotyledons	Oh and Komatsu (2015)
Proteomes	V2_Vegetative stage	Leaf fresh weight, leaf dry weight, leaf turgid weight, chlorophyll content chlorophyll content data	Mutava et al. (2015)
Proteomes	Vegetative stage	Root and leaf samples	Kazemi Oskuei et al. (2017)

*qRT-PCR, quantitative reverse transcription polymerase chain reaction; RT-PCR, reverse transcription polymerase chain reaction.*

### Meta-Analysis and Gene Prioritization

There are three steps in big data meta-analysis of FTgenes in soybean, including scoring, weighting, and ranking (Figure 1). Different scoring schemes were set to score test genes for each data source (Supplementary Table 1). In the association mapping data platform, the scores of 117 genes were between 4.01 and 10. In the linkage mapping data platform, 296 genes were scored between 1 and 10. In the gene expression data platform, the scores of 47,227 genes ranged between 1 and 6.45. In the pathway regulation data platform, the scores of 2,014 genes were between 5 and 6. In the PPIN data platform, the scores of 47,931 genes were between 1 and 6. In the networks data platform, the scores of 59 genes were ranged from 0 to 3. In the proteomes data platform, the scores of 376 genes were between 1.3 and 10.

In terms of weighting and weighted score (Supplementary Table 2), first of all, the range of weighting in the association mapping data platform is between 0 and 3. In the linkage mapping data platform, the range of weighting is between 0 and 4. In the gene expression and networks data platforms, the ranges of weighting were between 0 and 6. In pathway regulation and proteomes data platforms, the range of weighting is between 0 and 3. In the PPIN data platform, the range of weighting is between 0 and 2. Finally, after weighting by the impact factor, the weighted score of 36,705 flooding tolerance test genes was between 11.28 and 80.41. Furthermore, to identify FTgenes, a total of 28 test genes that passed the criteria were selected as the core genes (Supplementary Tables 4, 5).

**FIGURE 2:**
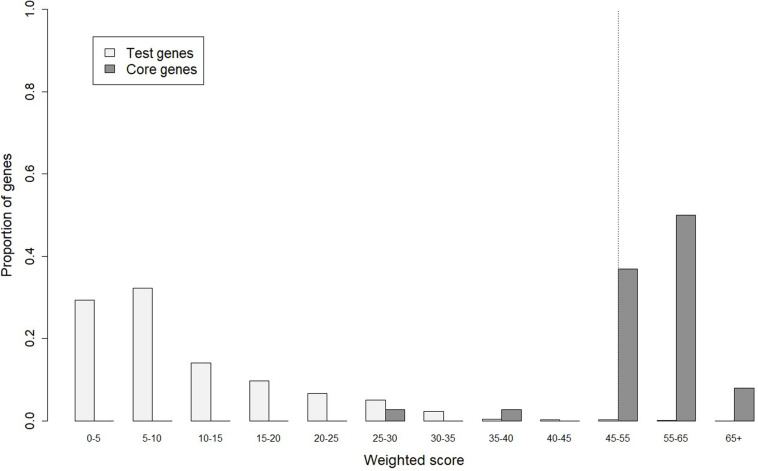
The optimal cut-off score in separating distributions of weighted scores between the test genes and the core genes.

**FIGURE 3:**
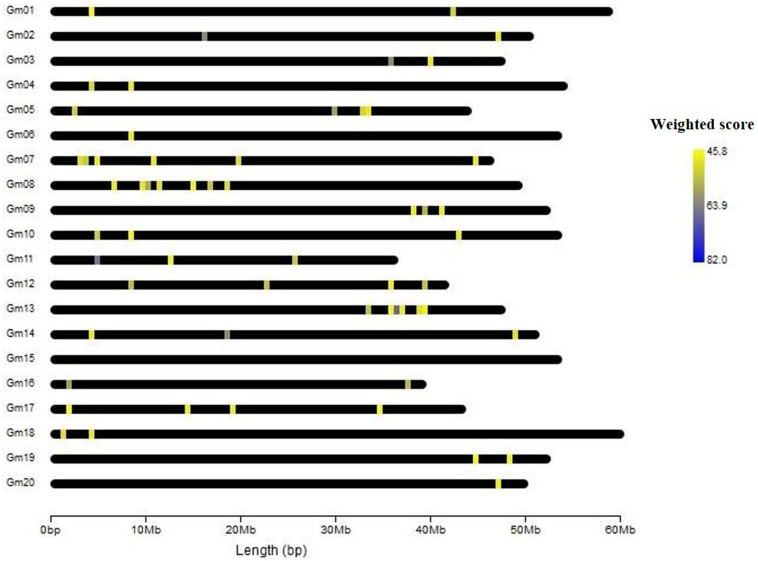
The location of 83 FTgenes on soybean genome map.

In gene prioritization, after calculating the weighted score for 36,705 test genes and 28 core genes, we compared the distribution of these two data sets. A trivial separation was observed between the test gene set and core gene set at a cutoff score of 45 (Figure 2), and a total of 83 genes were chosen as FTgenes (Table 3). The physical location and numbers of 83 FTgenes in the soybean genome are shown in Figure 3 and Supplementary Figure 2. Among them, chromosome 13 (14 FTgenes), 8 (13 FTgenes), and 7 (9 FTgenes) contain the most FTgenes. However, there are no FTgenes located on chromosomes 15 and 17.

**TABLE 3:** Eighty-three prioritized FTgenes (weighted score ≥ 45).

**Gene**	**Weighted score**	**Flooding treatment**	**Gene**	**Weighted score**	**Flooding treatment**
*Glyma.13g243800*	81.99	Submergence, waterlogging	*Glyma.08g119200*	49.19	Submergence, waterlogging
*Glyma.13g244000*	80.41	Submergence, waterlogging	*Glyma.14g202300*	49.00	Submergence
*Glyma.11g055700*	66.24	Submergence	*Glyma.08g119600*	48.65	Submergence, waterlogging
*Glyma.13g244100*	63.75	Submergence, waterlogging	*Glyma.08g119000*	48.10	Submergence, waterlogging
*Glyma.13g243700*	62.84	Submergence, waterlogging	*Glyma.13g251100*	48.05	Submergence
*Glyma.14g121200*	62.81	Submergence	*Glyma.10g180800*	48.02	Submergence
*Glyma.03g132700*	62.81	Submergence	*Glyma.08g083300*	47.81	Submergence
*Glyma.11g179300*	62.81	Submergence	*Glyma.14g049000*	47.81	Submergence
*Glyma.02g148200*	62.81	Submergence	*Glyma.07g105700*	47.81	Submergence
*Glyma.12g094100*	62.81	Submergence	*Glyma.12g093100*	47.81	Submergence
*Glyma.13g243600*	61.21	Submergence, waterlogging	*Glyma.09g172500*	47.81	Submergence
*Glyma.13g243900*	61.00	Submergence, waterlogging	*Glyma.02g268200*	47.81	Submergence
*Glyma.05g108900*	59.81	Submergence, waterlogging	*Glyma.03g173300*	47.81	Submergence
*Glyma.16g018500*	59.81	Submergence, waterlogging	*Glyma.18g042100*	47.81	Submergence
*Glyma.07g036400*	59.11	Submergence, waterlogging	*Glyma.17g174500*	47.81	Submergence
*Glyma.08g128500*	58.40	Submergence, waterlogging	*Glyma.11g181200*	47.81	Submergence
*Glyma.08g128100*	58.19	Submergence, waterlogging	*Glyma.06g100900*	47.81	Submergence
*Glyma.10g048000*	57.16	Submergence, waterlogging	*Glyma.05g128200*	47.81	Submergence
*Glyma.09g153900*	56.30	Submergence	*Glyma.07g049900*	47.81	Submergence
*Glyma.16g204600*	55.96	Submergence	*Glyma.04g092100*	47.81	Submergence
*Glyma.12g150500*	54.95	Submergence	*Glyma.17g158100*	47.81	Submergence
*Glyma.13g208000*	54.63	Submergence	*Glyma.07g253700*	47.81	Submergence
*Glyma.08g199800*	54.11	Submergence	*Glyma.09g149200*	47.81	Submergence
*Glyma.12g222400*	53.87	Submergence	*Glyma.20g218100*	47.81	Submergence
*Glyma.10g048100*	53.25	Submergence, waterlogging	*Glyma.12g187400*	47.81	Submergence
*Glyma.07g036300*	52.67	Submergence, waterlogging	*Glyma.08g119100*	47.64	Submergence, waterlogging
*Glyma.10g047800*	52.63	Submergence, waterlogging	*Glyma.11g180500*	47.52	Submergence
*Glyma.10g048200*	52.48	Submergence, waterlogging	*Glyma.08g119400*	47.29	Submergence, waterlogging
*Glyma.05g123900*	52.14	Submergence	*Glyma.13g279900*	47.09	Submergence
*Glyma.01g118000*	52.09	Submergence	*Glyma.19g213300*	46.87	Submergence
*Glyma.07g036200*	51.70	Submergence, waterlogging	*Glyma.08g176300*	46.85	Submergence
*Glyma.05g124000*	51.54	Submergence	*Glyma.08g119500*	46.70	Submergence, waterlogging
*Glyma.07g036100*	51.13	Submergence, waterlogging	*Glyma.13g234500*	46.47	Submergence
*Glyma.04g044900*	50.81	Submergence	*Glyma.10g073600*	46.36	Submergence
*Glyma.08g218600*	50.81	Submergence	*Glyma.13g251300*	46.20	Submergence
*Glyma.13g250400*	50.80	Submergence	*Glyma.13g250300*	46.13	Submergence
*Glyma.07g032900*	50.39	Submergence, waterlogging	*Glyma.01g037200*	46.13	Submergence
*Glyma.10g047900*	50.24	Submergence, waterlogging	*Glyma.19g174200*	46.00	Submergence
*Glyma.13g270100*	50.13	Submergence	*Glyma.11g149900*	45.96	Submergence
*Glyma.07g153100*	50.03	Submergence	*Glyma.17g020600*	45.85	Submergence
*Glyma.18g009700*	49.70	Submergence	*Glyma.17g205000*	45.81	Submergence
*Glyma.08g139100*	49.57	Submergence, waterlogging			

### Evaluation of Prioritized Genes

To evaluate the prioritized FTgenes, we compared two sets of FTgenes using a weighted scheme and an unweighted scheme (Supplementary Table 6). Table 4 showed the validation results. First, the 83 weighted FTgenes had significantly smaller *P*-values (higher FCs) than 100,000 random sets (3, 6, and 12 h: *P*-values <1×10^–5^, 24 h: *P*-value <0.01) using an independent whole genome RNA-seq database (Lin et al., 2019). However, the 83 unweighted FTgenes had significantly smaller *P*-values (higher FCs) than random sets at only 3 h and 6 h (*P*-values <0.05). The same situation was also observed in the intersection set (74 out of 83 genes, 89.16%) between weighted FTgenes and unweighted FT genes (Supplementary Table 10). Interestingly, we found that the set of FT genes^*w\nw*^ had significantly smaller *P*-values (higher FCs) than 100,000 random sets at 3, 6, and 12 h (*P*-values < 0.05), but the set of FTgenes^*nw\w*^ showed no significant difference at 3, 6, 12, and 24 h. This indicates that the set of FTgenes^*w\nw*^ may play roles in regulating mechanisms responding to flooding stress, suggesting that the use of a weighted scheme would provide informative and meaningful results.

**TABLE 4:** The results of comparing fold change after 3, 6, 12, and 24 hours of flooding treatment between the prioritized set and the random sets using the Wilcoxon rank-sum test.

**Method**	**Number of candidate genes**	***P*-value**			
		**3 h**	**6 h**	**12 h**	**24 h**
FTgenes (weighted)	83	<1×10^–5^	<1×10^–5^	<1×10^–5^	0.01
FTgenes (unweighted)	83	0.01	0.04	0.12	0.90
FTgenes (weighted ∩ unweighted)^a^	74	0.01	0.04	0.13	0.87
FTgenes (weighted\unweighted)^b^	9	0.01	0.01	0.05	0.43
FTgenes (unweighted\weighted)^c^	9	1.00	1.00	1.00	1.00
SoyNet^d^	83	0.60	0.03	0.35	0.99
Random forest	83	<1×10^–5^	<1×10^–5^	<1×10^–5^	<1×10^–5^

*^*a*^FTgenes (weighted ∩ unweighted) represents the intersection of weighted genes and unweighted genes.*

*^*b*^FTgenes (weighted\unweighted) represents the difference of unweighted FTgenes from weighted FTgenes.*

*^*c*^FTgenes (unweighted\weighted) represents the difference of weighted FTgenes from unweighted FTgenes.*

*^*d*^SoyNet represents the network analysis method used in Kim et al. (2017).*

The whole genome RNA-seq database was also used to evaluate 83 prioritized genes identified by the SoyNet network method (Kim et al., 2017). The results showed that there was no significant difference in FC between the prioritized set and 100,000 random sets. The proportion of *P*-value less than 0.05 does not exceed 81% in the four time points (Table 4). On the other hand, to compare the random forest method with our prioritized algorithm, the modified random forest method was conducted to satisfy our data format (Zhai et al., 2016). As a result, a total of 83 prioritized genes (ranked in the top 83 genes) were also identified based on the modified random forest method. Of which, the 83 prioritized genes were the same as our prioritized 83 FTgenes identified via an algorithm. The result suggested that the performance for our algorithm is as good as the random forest method, indicating our resulting 83 FTgenes are precise and reliable, which means our 83 weighted FTgenes had a higher chance to be involved in the physiological mechanism of flooding tolerance in soybean.

### Gene-Set Enrichment Analysis

To understand the physiological mechanisms of 83 FTgenes in soybean under flooding tolerance, we used the GO enrichment tool for gene-set enrichment analysis through SoyBase website. As shown in Table 5, the 83 FTgenes are significantly enriched in seven biological processes and molecular functions (*P*_*adjusted*_ – value <0.05). Our results suggested that the 83 FTgenes were significantly enriched in ethylene biosynthetic process, abscisic acid (ABA) biosynthetic process, nuclear-transcribed mRNA poly(A) tail shortening, glucan biosynthetic process, ethylene mediated signaling pathway, phosphorylation, and response to hypoxia.

**TABLE 5:** Gene-set enrichment analysis using 83 FTgenes.

**# GO annotation**	**Category**	**# FTgenes/# genes in pathway**	**Adjusted *P*-value^*a*^**
Ethylene biosynthetic process	Biological process	18/270	2.57×10^–11^
Abscisic acid (ABA) biosynthetic process	Biological process	5/53	3.63×10^–3^
Nuclear-transcribed mRNA poly(A) tail shortening	Biological process	3/11	6.94×10^–3^
Glucan biosynthetic process	Biological process	3/15	1.88×10^–2^
Ethylene mediated signaling pathway	Biological process	9/311	2.82×10^–2^
Phosphorylation	Biological process	5/84	3.34×10^–2^
Response to hypoxia	Biological process	7/195	4.19×10^–2^

*^*a*^Analysis was conducted using GO annotation database. Adjusted *P*-values were computed using the Bonferroni correction method.*

## Discussion

In the present study, we proposed a comprehensive framework that consists of bioinformatics big data mining, meta-analysis, and a gene prioritization algorithm to prioritize 83 FTgenes from 36,705 test genes set collected from multidimensional data platforms. We collected bioinformatics information including trait index, genetic data (SNP, gene, SSR, loci, and QTLs), variety, biochemical and statistical value (*P*-value, LOD, FC, score, *R*^2^, and keyword hits). The impact factors of journal articles were also collected for determining the reliability and quality of data. Our data was collected from a variety of countries, using different methods, plant materials, and genotype data, which is a diverse and informative database. There were three strengths to our gene prioritization method. First, we used quality control in big data collection to reduce the influence of noises effectively. Second, regardless of the positivity and negativity of the genotype data, we aimed to minimize the impact of publication bias. Third, we checked and unified gene aliases to avoid overestimations or underestimations that may occur in the calculation of the weighted score. Therefore, 83 FTgenes were found to have good characteristics of a more comprehensive, higher accuracy, and less bias and noise. Compared with traditional methods, this gene prioritization algorithm is more informative.

In the process of collecting bioinformatics big data, we found that GWAS had not been popular in the field of flooding tolerance in soybean, only two journal articles were found in the GWAS data platform (Table 1) (Wu et al., 2019; Yu et al., 2019). GWAS enables us to select the molecular markers that are significantly related to target traits. However, the main limitations of GWAS are low effect size and spurious associations. To cope with these problems, our research collected and integrated flooding tolerance candidate genes from different data sources and attempted to find high-ranking genes with a gene prioritization algorithm. Generally, high-ranking FTgenes should be reported in multiple data platforms, suggesting more potential that these are related to flooding tolerance compared to other genes. In our framework of analytic strategy, we can effectively reduced the chance of false positive results and increased the effect size that GWAS may encounter (Tam et al., 2019). In text mining, we searched the title and abstract of keywords (crop, gene, trait) with structured query language and R programming language, and found no results. We searched the full text alternatively, but most of the results were still irrelevant to the target. Therefore, in our opinion, text mining was not suitable for searching in the plant field at present.

The expression level of *Glyma.04g240800*, one of the 28 core genes, was repeatedly used to index the response of flooding stress of soybean in previous studies. *Glyma.04g240800* is one of the alcohol dehydrogenase (ADH) which participated in flooding tolerance. Flooding causes oxygen deprivation and forthwith activates anaerobic respiration in soybean. ADH reduces NAD^+^ to NADH in glycolysis, which is the first step of anaerobic respiration (Russell et al., 1990; Komatsu et al., 2009; Tucker et al., 2011; Nakayama et al., 2014; Song et al., 2018).

Of the 83 FTgenes, chromosome 13 contains the most FTgenes (Supplementary Figure 2), which reflects the results of a previous study (Yu et al., 2019). In chromosome 13, six FTgenes (*Glyma.13g243800*, *Glyma.13g244000*, *Glyma.13g244100*, *Glyma.13g243700*, *Glyma.13g243600*, and *Glyma.13g243900*) are in the top 12. Furthermore, the SNP marker QTN13 was reported to be remarkably related to flooding resistance (Yu et al., 2019), and these six genes are located within a 1.0 Mb region where QTN13 has extended the region of 500 kb upstream and downstream on both sides. The third-ranked FTgenes *Glyma.11g055700* was reported to show significant performance under flooding conditions (*P*-value = 0.00005) (Chen et al., 2016), and also reported participating in the ABA biosynthetic process in SoyBase. The 29th FTgene *Glyma.05g123900* was reported on four data platforms, which were gene expression, pathway regulation, PPIN, and proteomes. Moreover, this gene was also reported to show significant protein expression under flooding conditions (*P-*value = 5.57×10^–10^) and participated in the MAPK signaling pathway and plant pathogen interaction regulation pathway (Lin et al., 2019).

**FIGURE 4:**
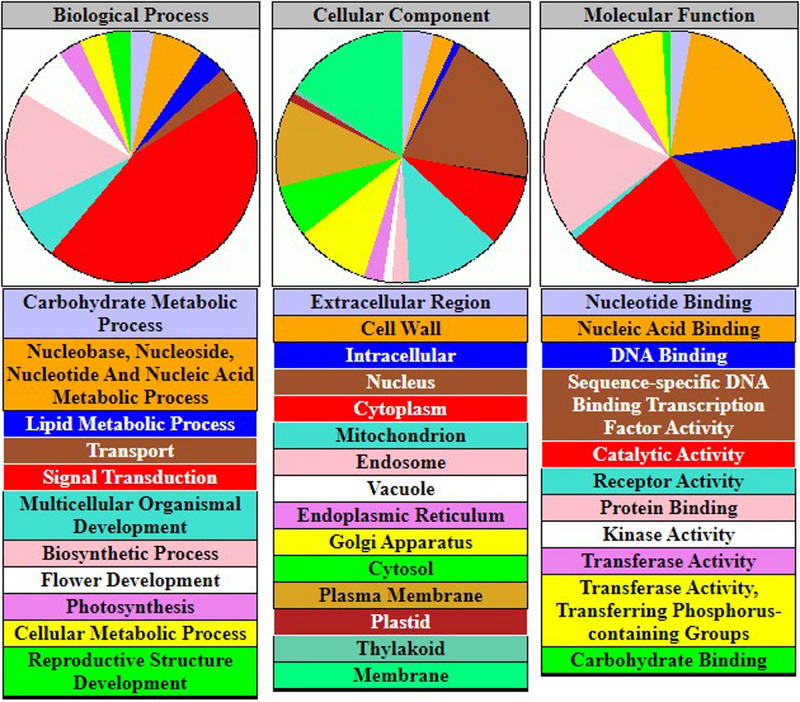
The gene-set enrichment analysis of 83 FTgenes.

In gene-set enrichment analysis, we found that seven GO pathways are significantly involved in the relevant mechanisms of flooding tolerance (Table 5 and Figure 4). GO:0009688 was the ABA biosynthetic process. Previous studies indicated that the concentration of ABA in hypocotyls will gradually decrease if soybeans are subjected to flooding stress, thereby leading to the growth of secondary aerenchyma (Shimamura et al., 2015). GO:0001666 is the pathway that responds to hypoxia. Flooding causes hypoxia in plant roots and induces hypoxia-related regulatory pathways, thus, it is intuitive that GO:0001666 was selected. GO:0009693 and GO:0009873 are the ethylene biosynthetic process and ethylene-activated signaling pathways, respectively. Yin et al. (2014) showed that the fresh weight of waterlogged soybean plants with ethylene application was significantly higher than the control one. This indicates that the presence of ethylene can help soybean plants resist flooding stress. In addition, the pathway of ethylene biosynthesis had been determined to be significantly related to the response of plants to flooding stress in previous studies (Morgan and Drew, 1997; Kim et al., 2017). The GO results above confirm that all of the 83 FTgenes we identified are reliable.

We compared 83 FTgenes with 83 equal weight prioritized genes for validation and found that there were 74 genes (89.16%) overlapping in both gene-sets (Supplementary Table 6). According to Fuller and Hester (1999), we speculated that it was owing to the existence of large sample outliers (LSO) in our big data meta-analysis. LSO will reduce the bias of the unweighted method, thereby narrowing the ranking of weighted and unweighted prioritized genes. Furthermore, three independent databases were also used for verification. In the first database, we included 27 SSR markers associated with flooding tolerance in soybean from SoyBase (Cregan et al., 1999; Cornelious et al., 2005; Githiri et al., 2006; Sayama et al., 2009; Rizal and Karki, 2011). We extended and selected the region 20 kb upstream and downstream of the 27 SSR markers on both sides by gene mapping. As a result, 87 genes were discovered (Supplementary Table 7). Only six genes overlapped between these 87 genes and the 83 FTgenes (*Glyma.08g119000*, *Glyma.08g119100*, *Glyma.08g119200*, *Glyma.08g119400*, *Glyma.08g119500*, and *Glyma.08g139100*). Subsequently, we found that the GO pathways which involved these 87 genes were not significantly associated with flooding tolerance. It is likely that the genes located at the region 20 kb upstream and downstream of the 27 SSR markers contain genes that were not related to flooding tolerance.

In another database, we compared 83 FTgenes with 59 prioritized genes which were identified by Kim et al. (2017) (Supplementary Table 8). We found that 16 genes (*Glyma.01g118000*, *Glyma.02g148200*, *Glyma.03g132700*, *Glyma.07g153100*, *Glyma.08g199800*, *Glyma.09g153900*, *Glyma.11g149900*, *Glyma.11g179300*, *Glyma.12g094100*, *Glyma.12g150500*, *Glyma.12g222400*, *Glyma.13g208000*, *Glyma.13g270100*, *Glyma.14g121200*, *Glyma.16g204600*, and *Glyma.18g009700*) overlapped in both gene-sets. The other 67 FTgenes without overlapping, were from the association data platform and pathway regulation data platform. The SoyNet database constructed by Kim et al. (2017) did not include these two data platforms. In the other database, we compared 83 FTgenes with the 117 prioritized genes identified by Yu et al. (2019) (Supplementary Table 9). Soon we found that 23 genes (*Glyma.13g243800*, *Glyma.13g244000*, *Glyma.11g055700*, *Glyma.13g244100*, *Glyma.13g243700*, *Glyma.13g243600*, *Glyma.13g243900*, *Glyma.07g036400*, *Glyma.08g128500*, *Glyma.08g128100*, *Glyma.10g048000*, *Glyma.10g048100*, *Glyma.07g036300*, *Glyma.10g047800*, *Glyma.10g048200*, *Glyma.07g036200*, *Glyma.07g036100*, *Glyma.13g250400*, *Glyma.10g047900*, *Glyma.14g202300*, *Glyma.13g251100*, *Glyma.13g251300*, and *Glyma.13g250300*) overlapped in both gene-sets. Comparing the fold change of these 117 prioritized genes with 100,000 bootstrap random groups by using the Wilcoxon rank-sum test, we discovered that their fold change did not show a significant difference (*P*-value >0.05). After validating the three independent databases above, we confirmed that our multi-data platforms are superior to a single data platform in terms of the accuracy of identifying FTgenes.

There are six FTgenes (*Glyma.13g243600*, *Glyma.13g243700*, *Glyma.13g243800*, *Glyma.13g243900*, *Glyma.13g244000*, and *Glyma.13g244100*) located in the same region in chromosome 13. However, the six FTgenes were reported in four different data platforms, including GWAS, linkage mapping, gene expression, and PPIN. Of which only linkage mapping data platform reported a QTL. Although the six FTgenes were significantly reported in several data platforms using various experiments or methods, further validation using an independent sample is needed to access which are casual genes or whether they are gene clusters. Another way of finding a casual gene (or an index SNP) in a QTL is to apply an LD-based clumping association test (Marees et al., 2018).

The similarities and differences of molecular mechanisms and responses to submergence and waterlogging have been studied in a wide range of species (Voesenek and Bailey-Serres, 2015). Both types of flooding stress limit oxygen availability in plant cells and produce hypoxia (<21% O_2_) (Sasidharan et al., 2017). There are two survival strategies, low-O_2_ escape syndrome (LOES) and low-O_2_ quiescence syndrome (LOQS), for flood-tolerant plants (Bailey-Serres and Voesenek, 2008, 2010; Voesenek and Bailey-Serres, 2013). The key responses for root waterlogging include the formation of aerenchyma and barriers in adventitious roots to avoid oxygen loss (Mustroph, 2018), which involves ethylene, reactive oxygen species (ROS), and hormonal signaling pathways including ABA and gibberellin (Voesenek and Bailey-Serres, 2015). The key responses for submergence include escape by elongation of aerial organs (LOES strategy for partial submergence), quiescence of metabolism and growth, protection of meristems or organs (Bailey-Serres and Voesenek, 2008, 2010; Bailey-Serres et al., 2012) (LOQS strategy for prolonged complete submergence), which involve signaling of ethylene, reduced light, low O_2_, nitric oxide, and ROS (Voesenek and Bailey-Serres, 2015). Our results in gene-set enrichment analysis (Table 5) are based on 83 prioritized FTgenes supported previous reported mechanisms or pathways including thylene biosynthetic process, ABA biosynthetic process, ethylene mediated signaling pathway, phosphorylation, and response to hypoxia. We conducted pathway analysis based on SUMSTAT with 10,000 permutations (Tintle et al., 2009), using 27 FTgenes that were identified to be associated or responded to both types of flooding stress (Table 3). Interestingly, the 27 FTgenes were significantly enriched with N-terminal protein myristoylation, response to hypoxia, defense response, secondary cell wall biogenesis, and biosynthetic process (*P*-values <1×10^−4^; data not shown), which again echo previously reported results reviewed by Voesenek and Bailey-Serres (2015).

There were two limitations to our study. The deficiency of a verified experiment of 83 FTgenes, e.g., RNA-seq transcriptome profiling and qRT-PCR, was the first limitation. Thus, the gene expression database from Lin et al. (2019) was adopted to evaluate the 83 FTgenes. We then obtained a significant difference between the prioritized set and the random set. In addition, 83 FTgenes were verified to be more reliable and robust by using three databases (GWAS, SoyNet, and SoyBase). The other limitation was that potential problems may exist in the results of previous studies, including noises and biases that affect our final prioritized results. To take over this problem, our big data meta-analysis and gene prioritization method can remove the genes with spurious associations. We are looking into the possibility of minimizing the effect of noises and biases from previous studies.

## Conclusion

To the best of our knowledge, this study is the first to report prioritized FTgenes for soybean. We introduced a comprehensive framework to integrate and prioritize diverse genetic data collected from multiple dimensional data sources to search for important genes that are highly connected to flooding tolerance or responding to stress. In the present study, a total of 83 FTgenes were prioritized, based on their magnitude of association or expression change, from a 36,705 test genes pool of flooding tolerance in soybean. These FTgenes were significantly enriched with a response to hypoxia, ethylene, ABA, and glucan biosynthetic process pathways, which play an important role in the biosynthesis of plant hormones in soybean.

These results provide a basis for breeders to design efficient markers near or within the target locus of the FTgenes, and then marker-assisted selection can be applied to introduce FTgenes into the genome of commercial cultivars, such that these cultivars will be characterized by the ability to adapt to stress caused by flooding. The proposed analytic framework applied in the present study provides a shortcut to overcome a challenge in identifying the most promising genes from a large candidate-gene pool for agricultural traits of interest.

## Data Availability Statement

The datasets presented in this study can be found in online repositories. The names of the repository/repositories and accession number(s) can be found in the article/Supplementary Material.

## Author Contributions

C-FK: study conception and design. M-CL, L-HJ, and C-FK: acquisition of data. C-FK and M-CL: analysis and interpretation of data. M-CL, C-FK, Z-YL, L-HJ, and Y-SL: draft and manuscript revision. All authors read and approved the final manuscript.

## Conflict of Interest

The authors declare that the research was conducted in the absence of any commercial or financial relationships that could be construed as a potential conflict of interest.

## Footnotes

1 https://www.ncbi.nlm.nih.gov/

2 https://www.airitilibrary.com/

3 http://www.pubmedchina.com/

4 https://soybase.org/dlpages/#annot

5 http://plantregmap.cbi.pku.edu.cn/download.php#networks

6 https://phytozome.jgi.doe.gov/pz/portal.html

7 https://soybase.org/correspondence/index.php

8 www.inetbio.org/soynet

## Supplementary Material

Supplementary Figure 1 The principle of gene mapping.

Supplementary Figure 2 Distribution of the quantity of 83 FTgenes on each soybean chromosome.

Supplementary Table 1 The scoring scheme of test genes from different data sources.

Supplementary Table 2 The range of impact factors with corresponding weight.

Supplementary Table 3 Summary results of big data mining of flooding tolerance in soybean.

Supplementary Table 4 The sources and association information for the five core genes selected from previous studies.

Supplementary Table 5 The sources and association information for the 23 core genes selected from multiple platforms.

Supplementary Table 6 Comparison of weighted and unweighted FTgenes by impact factor.

Supplementary Table 7 Gene mapping results between ± 20 kb of 27 SSR molecular marker in previous studies.

Supplementary Table 8 Fifty-nine soybean flooding tolerance prioritized genes identified in Kim et al. (2017).

Supplementary Table 9 The 117 genes mapped from GWAS data. Detailed information can be found in Yu et al. (2019).

Supplementary Table 10 The list of 74 overlapped FTgenes between 83 weighted genes and 83 unweighted genes.

Supplementary Material 1 Raw data of genomic big data mining from multidimensional data platforms.
