# Supplementary material for: Prioritization and Evaluation of Flooding Tolerance Genes in Soybean [Glycine max (L.) Merr.]
Source: Front Genet. 2021 Jan 27;11:612131. doi: 10.3389/fgene.2020.612131 (PMC7873447; doi:10.3389/fgene.2020.612131)
Supplement: Supplementary Figure 1 — The principle of gene mapping. [file Table_2.DOCX]

**Supplementary Table 1.** The score scheme of test genes from different data sources.

| **Data source** | **Scoring criteria** | **Score range** |
| --- | --- | --- |
| Association mapping (includes GWAS) | $S_{p-value}=\left\{ \begin{aligned} -{log}_{10}\left( P-value \right), if P-value\geq1\times{10}^{-8} \\ 9, if 1\times{10}^{-10}\leq P-value<1\times{10}^{-8} \\ 10, if P-value< 1\times{10}^{-10} \end{aligned} \right.$  $S_{\mathrm{association}}=I_{P-value\geq1\times{10}^{-8}}*\left( -{log}_{10}\left( P-value \right) \right)+$ $I_{1\times{10}^{-10}\leq P-value<1\times{10}^{-8}}*9+$ $I_{P-value\geq1\times{10}^{-10}}*10$ | [0, 10] |
| Linkage mapping | $S_{p-value}=\left\{ \begin{aligned} -{log}_{10}\left( P-value \right), if P-value\geq1\times{10}^{-8} \\ 9, if 1\times{10}^{-10}\leq P-value<1\times{10}^{-8} \\ 10, if P-value< 1\times{10}^{-10} \end{aligned} \right.$  $S_{LOD}=\left\{ \begin{aligned} [LOD], if LOD\leq8 \\ 9, if 8<\mathrm{LOD}\leq10 \\ 10, if LOD>10 \end{aligned} \right.$  $S_{linkage}=I_{LOD\leq8}*LOD+I_{8<LOD\leq10}*9+I_{LOD>10}*10$  $S_{\mathrm{linkage}} = Max(S_{LOD}, S_{P-value})$ | [0, 10] |
| Gene expression | $S_{p-value}=\left\{ \begin{aligned} -{log}_{10}\left( P-value \right), if P-value\geq1\times{10}^{-8} \\ 9, if 1\times{10}^{-10}\leq P-value<1\times{10}^{-8} \\ 10, if P-value< 1\times{10}^{-10} \end{aligned} \right.$  $S_{gExp}=\left\{ \begin{aligned} Max\{S_{P-value}, 1\}, if FC missing \\ Min\left\{ \frac{\left\vert FC \right\vert}{2},1.5 \right\}*Max\left\{ S_{P-value},1 \right\},if FC exists \end{aligned} \right.$ | [0, 15] |
| Regulatory pathway | $S_{pathway}=\left\{ \begin{aligned} 6, if reference \geq7 \\ 5,if pathway in score 6 contains \\ keywords regulation/mediate \\ 4,\mathrm{if} 3\leq\mathrm{reference}\leq6 \\ 3, if pathway in score 4 contains \\ keywords regulation/mediate \\ 2,if reference\leq2 \\ 1,if pathway in score 2 contains \\ keywords regulation/mediate \end{aligned} \right.$ | {1, 2, 3, 4, 5, 6} |
| Literature | $S_{\mathrm{literature}}=\left\{ \begin{aligned} 1,if hits on keywords (gene symbol+trait) \\ 0,if no hits were made \end{aligned} \right.$ | {0, 1} |
| PPIN | $S_{PPIN}=\left\{ \begin{aligned} 1, if degree<100 and cc<0.5 \\ 2,if degree<100 and cc\geq0.5 \\ 3,if degree\in[100, 1000) and cc<0.1 \\ 4, if drgree\in[100, 1000) &and cc\geq0.1 \\ 5,if drgree\geq1000 &and cc<0.01 \\ 6,if drgree\geq1000 &and cc\geq0.01 \end{aligned} \right.$ | {1, 2, 3, 4, 5, 6} |
| Proteomes | $S_{Proteomes}=\left\{ \begin{aligned} Max\{-{log}_{10}\left( P-value \right), 1/\vert1-r\vert\}, if r exists \\ \mathrm{Max}\{-{log}_{10}\left( P-value \right),if FC exists \end{aligned} \right.$ | [1, 6] |
| Network | $S_{Network}=\left\{ \begin{aligned} -{log}_{10}\left( P-value \right), if P-value exist \\ [\vert FC\vert], if \mathrm{FC}\mathrm{exists} \\ I_{[degree < 10]}+2*I_{\left[ degree in \left[ 10, 15 \right] \right]}+3*I_{\left[ \mathrm{degree}\geq16 \right]} \\ if degree exists \end{aligned} \right.$ | [1, 6] |
| Abbreviation: GWAS, genome-wide association study; LOD, logarithm of the odds; FC, fold change; PPIN, protein-protein interaction network; cc, cluster coefficient; r, correlation. I, indicator function. | | |

**Supplementary Table** **2.** The range of impact factor with the corresponding weight.

| Range of Impact Factor | Weight |
| --- | --- |
| IF^a^ ≧ 8 | 6 |
| 8 > IF ≧ 6 | 5 |
| 6 > IF ≧ 5 | 4 |
| 5 > IF ≧ 3 | 3 |
| 3 > IF ≧ 1 | 2 |
| 1 > IF > 0 | 1 |
| Abbreviation: IF, impact factor. | |

**Supplementary Table** **3.** Summary results of big data mining of flooding tolerance in soybean.

| **Data source** | **Methods** | **No. of genomic data** | | | | | **References** |
| --- | --- | --- | --- | --- | --- | --- | --- |
|  |  | **#SNP** | **#Gene** | **#SSR** | **#QTL** | **#Database** |  |
| Association mapping (includes GWAS) | Regression analysis, Pearson’s correlation coefficient test,  MLM & mrMLM | 79 | 0 | 0 | 0 | 0 | Wu *et al.* (2020), Yu *et al.* (2019), Ye *et al.* (2018) |
| Linkage mapping | Regression analysis | 0 | 0 | 66 | 0 | 0 | Nguyen *et al.* (2012), Rizal & Karki, (2011), Sayama *et al.* (2009), Githiri *et al.* (2006), Cornelious *et al.* (2005),Vantoai *et al.* (2001), Cregan *et al.* (1999) |
| Gene expression | qRT-PCR | 0 | 47,227 | 0 | 0 | 2 | Song *et al.* (2018), Nakayama *et al.* (2017), Chen *et al.* (2016), Syed *et al.* (2015), Nakayama *et al.* (2014), Valliyodan *et al.* (2014), Nishizawa *et al.* (2013), Nanjo *et al.* (2011), Komatsu *et al.* (2009) |
| Gene pathway regulatory | Spectrometry analysis | 0 | 2,014 | 0 | 0 | 1 | Yin *et al.* (2017), Hashiguchi and Komatsu (2016), Yin *et al.* (2016), Yin and Komatsu (2016), Yin and Komatsu (2015), Mustafa *et al.* (2015), Oh and Komatsu (2015), Khan *et al.* (2015), Yin *et al.* (2014), Valliyodan *et al.* (2014), Mustafa and Komatsu (2014), Nanjo *et al.* (2012), Khatoon *et al.* (2012), Nanjo *et al.* (2011), Nanjo *et al.* (2010) |
| Gene networks | gene co-expression network analysis, genome context methods | 0 | 59 | 0 | 0 | 0 | Kim *et al.* (2017) |
| PPIN | Interactome analysis | 0 | 47,931 | 0 | 0 | 1 | PlantRegMap |
| Homologous gene | Sequence alignment | 0 | 8,511 | 0 | 0 | 0 | Hwang *et al.* (2020), Giuntoil *et al.* (2017), White *et al.* (2017), Vashisht *et al.* (2016), Veen *et al.* (2016), Giuntoil *et al.* (2014), Im *et al.* (2014), Lee *et al.* (2011), Yang *et al.* (2014), Cho *et al.* (2012), Park *et al.* (2011) |
| Proteomics | Pearson’s correlation coefficient test | 0 | 376 | 0 | 0 | 0 | Kazemi Oskuei *et al.* (2017), Mutava *et al.* (2015), Oh *et al.* (2015), Nanjo *et al.* (2014) |
| Abbreviation: GWAS, genome-wide association study; PPIN, protein-protein interaction network; MLM, mixed linear model; mrMLM, multi-locus random-SNP effect mixed linear model; qRT-PCR, quantitative reverse transcription polymerase chain reaction. | | | | | | | |

**REFERENCES**

Chen, W., Yao, Q., Patil, G.B., Agarwal, G., Deshmukh, R.K., Lin, L., et al. (2016). Identification and comparative analysis of differential gene expression in soybean leaf tissue under drought and flooding stress revealed by RNA-Seq. *Front Plant Sci* 7**,** 1044. doi: 10.3389/fpls.2016.01044.

Cho, Y.H., Hong, J.W., Kim, E.C., and Yoo, S.D. (2012). Regulatory functions of SnRK1 in stress-responsive gene expression and in plant growth and development. *Plant Physiol* 158(4)**,** 1955-1964. doi: 10.1104/pp.111.189829.

Cornelious, B., Chen, P., Chen, Y., de Leon, N., Shannon, J.G., and Wang, D. (2005). Identification of QTLs underlying water-logging tolerance in soybean. *Molecular Breeding* 16(2)**,** 103-112. doi: 10.1007/s11032-005-5911-2.

Cregan, P.B., Jarvik, T., Bush, A.L., Shoemaker, R.C., Lark, K.G., Kahler, A.L., et al. (1999). An integrated genetic linkage map of the soybean genome. *Crop Sci* 39. doi: 10.2135/cropsci1999.3951464x.

Githiri, S.M., Watanabe, S., Harada, K., and and Takahashi, R. (2006). QTL analysis of flooding tolerance in soybean at an early vegetative growth stage. *Plant Breeding* 125**,** 613-618.

Giuntoli, B., Lee, S.C., Licausi, F., Kosmacz, M., Oosumi, T., van Dongen, J.T., et al. (2014). A trihelix DNA binding protein counterbalances hypoxia-responsive transcriptional activation in Arabidopsis. *PLoS Biol* 12(9)**,** e1001950. doi: 10.1371/journal.pbio.1001950.

Giuntoli, B., Shukla, V., Maggiorelli, F., Giorgi, F.M., Lombardi, L., Perata, P., et al. (2017). Age-dependent regulation of ERF-VII transcription factor activity in Arabidopsis thaliana. *Plant Cell Environ* 40(10)**,** 2333-2346. doi: 10.1111/pce.13037.

Hashiguchi, A., and Komatsu, S. (2016). Impact of post-translational modifications of crop proteins under abiotic stress. *Proteomes* 4(4). doi: 10.3390/proteomes4040042.

Hwang, J.H., Yu, S.I., Lee, B.H., and Lee, D.H. (2020). Modulation of Energy Metabolism Is Important for Low-Oxygen Stress Adaptation in Brassicaceae Species. *Int J Mol Sci* 21(5). doi: 10.3390/ijms21051787.

Im, J.H., Cho, Y.H., Kim, G.D., Kang, G.H., Hong, J.W., and Yoo, S.D. (2014). Inverse modulation of the energy sensor Snf1-related protein kinase 1 on hypoxia adaptation and salt stress tolerance in Arabidopsis thaliana. *Plant Cell Environ* 37(10)**,** 2303-2312. doi: 10.1111/pce.12375.

Kazemi Oskuei, B., Yin, X., Hashiguchi, A., Bandehagh, A., and Komatsu, S. (2017). Proteomic analysis of soybean seedling leaf under waterlogging stress in a time-dependent manner. *Biochim Biophys Acta Proteins Proteom* 1865(9)**,** 1167-1177. doi: 10.1016/j.bbapap.2017.06.022.

Khan, M.N., Sakata, K., and Komatsu, S. (2015). Proteomic analysis of soybean hypocotyl during recovery after flooding stress. *J Proteomics* 121**,** 15-27. doi: 10.1016/j.jprot.2015.03.020.

Khatoon, A., Rehman, S., Salavati, A., and Komatsu, S. (2012). A comparative proteomics analysis in roots of soybean to compatible symbiotic bacteria under flooding stress. *Amino Acids* 43(6)**,** 2513-2525. doi: 10.1007/s00726-012-1333-8.

Kim, E., Hwang, S., and Lee, I. (2017). SoyNet: a database of co-functional networks for soybean *Glycine max*. *Nucleic Acids Res* 45(D1)**,** D1082-D1089. doi: 10.1093/nar/gkw704.

Komatsu, S., Yamamoto, R., Nanjo, Y., Mikami, Y., Yunokawa, H., and Sakata, K. (2009). A comprehensive analysis of the soybean genes and proteins expressed under flooding stress using transcriptome and proteome techniques. *Journal of Proteome Research* 8**,** 4766-4778.

Lee, S.C., Mustroph, A., Sasidharan, R., Vashisht, D., Pedersen, O., Oosumi, T., et al. (2011). Molecular characterization of the submergence response of the Arabidopsis thaliana ecotype Columbia. *New Phytol* 190(2)**,** 457-471. doi: 10.1111/j.1469-8137.2010.03590.x.

Mansur, L.M., Orf, J.H., Chase, K., Jarvik, T., Cregan, P.B., and Lark, K.G. (1996). Genetic mapping of agronomic traits using recombinant inbred lines of soybean. *Crop Sci* 36. doi: 10.2135/cropsci1996.0011183X003600050042x.

Mustafa, G., and Komatsu, S. (2014). Quantitative proteomics reveals the effect of protein glycosylation in soybean root under flooding stress. *Front Plant Sci* 5**,** 627. doi: 10.3389/fpls.2014.00627.

Mustafa, G., Sakata, K., Hossain, Z., and Komatsu, S. (2015). Proteomic study on the effects of silver nanoparticles on soybean under flooding stress. *J Proteomics* 122**,** 100-118. doi: 10.1016/j.jprot.2015.03.030.

Mutava, R.N., Prince, S.J.K., Syed, N.H., Song, L., Valliyodan, B., Chen, W., et al. (2015). Understanding abiotic stress tolerance mechanisms in soybean: a comparative evaluation of soybean response to drought and flooding stress. *Plant Physiol Biochem* 86**,** 109-120. doi: 10.1016/j.plaphy.2014.11.010.

Nakayama, T.J., Rodrigues, F.A., Neumaier, N., Marcelino-Guimarães, F.C., Farias, J.R.B., de Oliveira, M.C.N., et al. (2014). Reference genes for quantitative real-time polymerase chain reaction studies in soybean plants under hypoxic conditions. *Genetics and Molecular Research* 13(1)**,** 860-871. doi: 10.4238/2014.February.13.4.

Nakayama, T.J., Rodrigues, F.A., Neumaier, N., Marcolino-Gomes, J., Molinari, H.B.C., Santiago, T.R., et al. (2017). Insights into soybean transcriptome reconfiguration under hypoxic stress: Functional, regulatory, structural, and compositional characterization. *PLoS One* 12(11)**,** e0187920. doi: 10.1371/journal.pone.0187920.

Nanjo, Y., Jang, H.Y., Kim, H.S., Hiraga, S., Woo, S.H., and Komatsu, S. (2014). Analyses of flooding tolerance of soybean varieties at emergence and varietal differences in their proteomes. *Phytochemistry* 106**,** 25-36. doi: 10.1016/j.phytochem.2014.06.017.

Nanjo, Y., Maruyama, K., Yasue, H., Yamaguchi-Shinozaki, K., Shinozaki, K., and Komatsu, S. (2011). Transcriptional responses to flooding stress in roots including hypocotyl of soybean seedlings. *Plant Mol Biol* 77(1-2)**,** 129-144. doi: 10.1007/s11103-011-9799-4.

Nanjo, Y., Skultety, L., Ashraf, Y., and and Komatsu, S. (2010). Comparative proteomic analysis of early-stage soybean seedlings responses to flooding by using gel and gel-free techniques. *Journal of Proteome Research* 9**,** 3989-4002.

Nanjo, Y., Skultety, L., Uvackova, L., Klubicova, K., Hajduch, M., and Komatsu, S. (2012). Mass spectrometry-based analysis of proteomic changes in the root tips of flooded soybean seedlings. *J Proteome Res* 11(1)**,** 372-385. doi: 10.1021/pr200701y.

Nguyen, V.T., Vuong, T.D., VanToai, T., Lee, J.D., Wu, X., Mian, M.A.R., et al. (2012). Mapping of quantitative trait loci associated with resistance to phytophthora sojae and flooding tolerance in soybean. *Crop Sci* 52(6)**,** 2481-2493. doi: 10.2135/cropsci2011.09.0466.

Nishizawa, K., Hiraga, S., Yasue, H., Chiba, M., Tougou, M., Nanjo, Y., et al. (2013). The synthesis of cytosolic ascorbate peroxidases in germinating seeds and seedlings of soybean and their behavior under flooding stress. *Biosci Biotechnol Biochem* 77(11)**,** 2205-2209. doi: 10.1271/bbb.130384.

Oh, M., and Komatsu, S. (2015). Characterization of proteins in soybean roots under flooding and drought stresses. *J Proteomics* 114**,** 161-181. doi: 10.1016/j.jprot.2014.11.008.

Park, H.Y., Seok, H.Y., Woo, D.H., Lee, S.Y., Tarte, V.N., Lee, E.H., et al. (2011). AtERF71/HRE2 transcription factor mediates osmotic stress response as well as hypoxia response in Arabidopsis. *Biochem Biophys Res Commun* 414(1)**,** 135-141. doi: 10.1016/j.bbrc.2011.09.039.

Rizal, G., and Karki, S. (2011). Alcohol dehydrogenase (ADH) activity in soybean [*Glycine max* (L.) Merr.] under flooding stress. *Electronic Journal of Plant Breeding* 2(1)**,** 50-57.

Sayama, T., Nakazaki, T., Ishikawa, G., Yagasaki, K., Yamada, N., Hirota, N., et al. (2009). QTL analysis of seed-flooding tolerance in soybean [*Glycine max* (L.) Merr.]. *Plant Sci* 176(4)**,** 514-521. doi: 10.1016/j.plantsci.2009.01.007.

Song, L., Valliyodan, B., Prince, S., Wan, J., and Nguyen, H. (2018). Characterization of the XTH gene family: New insight to the roles in soybean flooding tolerance. *International Journal of Molecular Sciences* 19(9). doi: 10.3390/ijms19092705.

Song, Q.J., Marek, L.F., Shoemaker, R.C., Lark, K.G., Concibido, V.C., Delannay, X., et al. (2004). A new integrated genetic linkage map of the soybean. *Theor Appl Genet* 109. doi: 10.1007/s00122-004-1602-3.

Syed, N.H., Prince, S.J., Mutava, R.N., Patil, G., Li, S., Chen, W., et al. (2015). Core clock, SUB1, and ABAR genes mediate flooding and drought responses via alternative splicing in soybean. *J Exp Bot* 66(22)**,** 7129-7149. doi: 10.1093/jxb/erv407.

Valliyodan, B., Van Toai, T.T., Alves, J.D., de Fatima, P.G.P., Lee, J.D., Fritschi, F.B., et al. (2014). Expression of root-related transcription factors associated with flooding tolerance of soybean [*Glycine max* (L.) Merr.]. *Int J Mol Sci* 15(10)**,** 17622-17643. doi: 10.3390/ijms151017622.

van Veen, H., Vashisht, D., Akman, M., Girke, T., Mustroph, A., Reinen, E., et al. (2016). Transcriptomes of Eight Arabidopsis thaliana Accessions Reveal Core Conserved, Genotype- and Organ-Specific Responses to Flooding Stress. *Plant Physiol* 172(2)**,** 668-689. doi: 10.1104/pp.16.00472.

VanToai, T.T., Martin, K.S.S., Chase, K., Boru, G., Schnipke, V., Schmitthenner, F.A., et al. (2001). Identification of a QTL associated with tolerance of soybean to soil waterlogging. *Crop Sci* 41**,** 1247-1252.

Vashisht, D., van Veen, H., Akman, M., and Sasidharan, R. (2016). Variation in Arabidopsis flooding responses identifies numerous putative "tolerance genes". *Plant Signal Behav* 11(11)**,** e1249083. doi: 10.1080/15592324.2016.1249083.

White, M.D., Klecker, M., Hopkinson, R.J., Weits, D.A., Mueller, C., Naumann, C., et al. (2017). Plant cysteine oxidases are dioxygenases that directly enable arginyl transferase-catalysed arginylation of N-end rule targets. *Nat Commun* 8**,** 14690. doi: 10.1038/ncomms14690.

Wu, C., Mozzoni, L.A., Moseley, D., Hummer, W., Ye, H., Chen, P., et al. (2019). Genome-wide association mapping of flooding tolerance in soybean. *Molecular Breeding* 40(1). doi: 10.1007/s11032-019-1086-0.

Yang, C.Y. (2014). Hydrogen peroxide controls transcriptional responses of ERF73/HRE1 and ADH1 via modulation of ethylene signaling during hypoxic stress. *Planta* 239(4)**,** 877-885. doi: 10.1007/s00425-013-2020-z.

Ye, H., Song, L., Chen, H., Valliyodan, B., Cheng, P., Ali, L., et al. (2018). A major natural genetic variation associated with root system architecture and plasticity improves waterlogging tolerance and yield in soybean. *Plant Cell Environ* 41(9)**,** 2169-2182. doi: 10.1111/pce.13190.

Yin, X., Hiraga, S., Hajika, M., Nishimura, M., and Komatsu, S. (2017). Transcriptomic analysis reveals the flooding tolerant mechanism in flooding tolerant line and abscisic acid treated soybean. *Plant Mol Biol* 93(4-5)**,** 479-496. doi: 10.1007/s11103-016-0576-2.

Yin, X., and Komatsu, S. (2015). Quantitative proteomics of nuclear phosphoproteins in the root tip of soybean during the initial stages of flooding stress. *J Proteomics* 119**,** 183-195. doi: 10.1016/j.jprot.2015.02.004.

Yin, X., and Komatsu, S. (2016). Nuclear proteomics reveals the role of protein synthesis and chromatin structure in root tip of soybean during the initial stage of flooding stress. *J Proteome Res* 15(7)**,** 2283-2298. doi: 10.1021/acs.jproteome.6b00330.

Yin, X., Nishimura, M., Hajika, M., and Komatsu, S. (2016). Quantitative proteomics reveals the flooding-tolerance mechanism in mutant and abscisic acid-treated soybean. *J Proteome Res* 15(6)**,** 2008-2025. doi: 10.1021/acs.jproteome.6b00196.

Yin, X., Sakata, K., and Komatsu, S. (2014). Phosphoproteomics reveals the effect of ethylene in soybean root under flooding stress. *J Proteome Res* 13(12)**,** 5618-5634. doi: 10.1021/pr500621c.

Yu, Z., Chang, F., Lv, W., Sharmin, R.A., Wang, Z., Kong, J., et al. (2019). Identification of QTN and candidate gene for seed-flooding tolerance in soybean [*Glycine max* (L.) Merr.] using genome-wide association study (GWAS). *Genes (Basel)* 10(12). doi: 10.3390/genes10120957.

**Supplementary Table** **4.** The sources and association information for the five core genes selected from review.

| Gene^a^ | # Data platform (≥4) | #{S_i_≥4} | weighteded score (>40) | Rank (top 2%) | References |
| --- | --- | --- | --- | --- | --- |
| *Glyma.04g240800* | Gene expression, PPIN, proteomics | 2 | 44.81 | 85 (0.23%) | Song *et al.* (2018), Nakayama *et al.* (2014), Tucker *et al.* (2011),  Komatsu *et al.* (2009) |
| *Glyma.11g123400* | Gene expression, PPIN | 1 | 29.81 | 596(1.6%) | Nanjo *et al.* (2011) |
| *Glyma.14g121200* | Gene expression, PPIN, proteomics, Networks | 2 | 62.81 | 6(0.016%) | Valliyodan *et al.* (2014), Komatsu *et al.* (2009) |
| *Glyma.07g264200* | Gene expression, PPIN, pathway regulatory | 1 | 36.02 | 278(0.76%) | Valliyodan *et al.* (2014) |
| *Glyma.03g223000* | Gene expression, PPIN, pathway regulatory, proteomics | 1 | 27.63 | 2187(5.96%) | Kim *et al.* (2017) |
| Abbreviation: #{S_i_≥4}, at least three or more data platforms having scores≥4.  ^a^Two inclusions are employed for the selection of core-gene set: (i) rank<top 2% (out of 36,697 genes), and (ii) number of data platforms≥4, combined scores>40 and at least three or more data platforms having scores≥4. | | | | | |

**REFERENCES**

Kim, E., Hwang, S., and Lee, I. (2017). SoyNet: a database of co-functional networks for soybean *Glycine max*. *Nucleic Acids Res* 45(D1)**,** D1082-D1089. doi: 10.1093/nar/gkw704.

Komatsu, S., Yamamoto, R., Nanjo, Y., Mikami, Y., Yunokawa, H., and Sakata, K. (2009). A comprehensive analysis of the soybean genes and proteins expressed under flooding stress using transcriptome and proteome techniques. *Journal of Proteome Research* 8**,** 4766-4778.

Nakayama, T.J., Rodrigues, F.A., Neumaier, N., Marcelino-Guimarães, F.C., Farias, J.R.B., de Oliveira, M.C.N., et al. (2014). Reference genes for quantitative real-time polymerase chain reaction studies in soybean plants under hypoxic conditions. *Genetics and Molecular Research* 13(1)**,** 860-871. doi: 10.4238/2014.February.13.4.

Nanjo, Y., Maruyama, K., Yasue, H., Yamaguchi-Shinozaki, K., Shinozaki, K., and Komatsu, S. (2011). Transcriptional responses to flooding stress in roots including hypocotyl of soybean seedlings. *Plant Mol Biol* 77(1-2)**,** 129-144. doi: 10.1007/s11103-011-9799-4.

Song, L., Valliyodan, B., Prince, S., Wan, J., and Nguyen, H. (2018). Characterization of the XTH gene family: New insight to the roles in soybean flooding tolerance. *International Journal of Molecular Sciences* 19(9). doi: 10.3390/ijms19092705.

Tucker, M.L., Murphy, C.A., and Yang, R. (2011). Gene expression profiling and shared promoter motif for cell wall-modifying proteins expressed in soybean cyst nematode-infected roots. *Plant Physiology* 156(1)**,** 319-329. doi: 10.1104/pp.110.170357.

Valliyodan, B., Van Toai, T.T., Alves, J.D., de Fatima, P.G.P., Lee, J.D., Fritschi, F.B., et al. (2014). Expression of root-related transcription factors associated with flooding tolerance of soybean [*Glycine max* (L.) Merr.]. *Int J Mol Sci* 15(10)**,** 17622-17643. doi: 10.3390/ijms151017622.

**Supplementary Table** **5.** The sources and association information for the 23 core genes selected from multiple platforms.

| **Gene** | **Score of association platform** | **Score of linkage analysis platform** | **Score of gene expression platform** | **Score of pathway regulatory platform** | **Score of PPIN platform** | **Score of proteomes platform** | **Score of networks platform** | **No. of data sources** | **Weighted score** |
| --- | --- | --- | --- | --- | --- | --- | --- | --- | --- |
| *Glyma.13g243800* | 30 | 27 | 20.99 | 0 | 4 | 0 | 0 | 4 | 81.99 |
| *Glyma.13g244000* | 30 | 27 | 19.41 | 0 | 4 | 0 | 0 | 4 | 80.41 |
| *Glyma.11g055700* | 18.95 | 0 | 25.29 | 18 | 4 | 0 | 0 | 4 | 66.24 |
| *Glyma.13g244100* | 30 | 27 | 2.75 | 0 | 4 | 0 | 0 | 4 | 63.75 |
| *Glyma.13g243700* | 30 | 27 | 1.84 | 0 | 4 | 0 | 0 | 4 | 62.84 |
| *Glyma.13g243600* | 30 | 27 | 0.2056 | 0 | 4 | 0 | 0 | 4 | 61.21 |
| *Glyma.05g108900* | 0 | 12 | 25.81 | 18 | 4 | 0 | 0 | 4 | 59.80 |
| *Glyma.16g018500* | 0 | 12 | 25.81 | 18 | 4 | 0 | 0 | 4 | 59.80 |
| *Glyma.07g036400* | 27 | 20.05 | 8.06 | 0 | 4 | 0 | 0 | 4 | 59.11 |
| *Glyma.08g128500* | 19.48 | 16.06 | 3.86 | 15 | 4 | 0 | 0 | 5 | 58.40 |
| *Glyma.08g128100* | 19.48 | 16.06 | 18.65 | 0 | 4 | 0 | 0 | 4 | 58.19 |
| *Glyma.10g048000* | 27 | 21.15 | 5.01 | 0 | 4 | 0 | 0 | 4 | 57.16 |
| *Glyma.05g123900* | 0 | 0 | 25.81 | 15 | 4 | 7.33 | 0 | 4 | 54.48 |
| *Glyma.10g048100* | 27 | 21.15 | 1.1 | 0 | 4 | 0 | 0 | 4 | 53.25 |
| *Glyma.05g124000* | 0 | 0 | 25.81 | 15 | 4 | 6.73 | 0 | 4 | 52.70 |
| *Glyma.07g036300* | 27 | 20.05 | 1.61 | 0 | 4 | 0 | 0 | 4 | 52.67 |
| *Glyma.10g047800* | 27 | 21.15 | 0.48 | 0 | 4 | 0 | 0 | 4 | 52.63 |
| *Glyma.10g048200* | 27 | 21.15 | 0.33 | 0 | 4 | 0 | 0 | 4 | 52.48 |
| *Glyma.07g036200* | 27 | 20.05 | 0.65 | 0 | 4 | 0 | 0 | 4 | 51.70 |
| *Glyma.07g036100* | 27 | 20.05 | 0.08 | 0 | 4 | 0 | 0 | 4 | 51.13 |
| *Glyma.10g047900* | 27 | 21.53 | 0.09 | 0 | 4 | 0 | 0 | 4 | 50.24 |
| *Glyma.07g031400* | 13.53 | 15.62 | 0.324 | 0 | 4 | 10.34 | 0 | 5 | 43.82 |
| *Glyma.02g224000* | 0 | 0 | 5.61 | 0 | 4 | 15 | 18 | 4 | 42.61 |

**Supplementary Table** **6.** Comparison of weighted and unweighted FTgenes by impact factor.

| **Weighted list** | **Score of association platform** | **Score of linkage analysis platform** | **Score of gene expression platform** | **Score of pathway regulatory platform** | **Score of PPIN platform** | **Score of proteomes platform** | **Score of networks platform** | **Weighted score** | **Unweighted list** | **Score of association platform** | **Score of linkage analysis platform** | **Score of gene expression platform** | **Score of pathway regulatory platform** | **Score of PPIN platform** | **Score of proteomes platform** | **Score of networks platform** | **Equal weight score** |
| --- | --- | --- | --- | --- | --- | --- | --- | --- | --- | --- | --- | --- | --- | --- | --- | --- | --- |
| *Glyma.13g243800* | 30 | 27 | 20.989 | 0 | 4 | 0 | 0 | 81.99 | *Glyma.13g243800* | 10 | 9 | 5.2472781 | 0 | 2 | 0 | 0 | 26.25 |
| *Glyma.13g244000* | 30 | 27 | 19.412 | 0 | 4 | 0 | 0 | 80.41 | *Glyma.13g244000* | 10 | 9 | 4.853088701 | 0 | 2 | 0 | 0 | 25.85 |
| *Glyma.11g055700* | 18.95 | 0 | 25.292 | 18 | 4 | 0 | 0 | 66.24 | *Glyma.13g244100* | 10 | 9 | 0.688173878 | 0 | 2 | 0 | 0 | 21.69 |
| *Glyma.13g244100* | 30 | 27 | 2.7527 | 0 | 4 | 0 | 0 | 63.75 | *Glyma.13g243700* | 10 | 9 | 0.458953183 | 0 | 2 | 0 | 0 | 21.46 |
| *Glyma.13g243700* | 30 | 27 | 1.8358 | 0 | 4 | 0 | 0 | 62.84 | *Glyma.13g243600* | 10 | 9 | 0.0514 | 0 | 2 | 0 | 0 | 21.05 |
| *Glyma.14g121200* | 0 | 0 | 25.806 | 0 | 4 | 15 | 36 | 62.81 | *Glyma.13g243900* | 10 | 9 | 0 | 0 | 2 | 0 | 0 | 21.00 |
| *Glyma.03g132700* | 0 | 0 | 25.806 | 0 | 4 | 15 | 36 | 62.81 | *Glyma.11g055700* | 6.316800003 | 0 | 6.322922691 | 6 | 2 | 0 | 0 | 20.64 |
| *Glyma.11g179300* | 0 | 0 | 25.806 | 0 | 4 | 15 | 36 | 62.81 | *Glyma.08g128500* | 6.493436351 | 5.35261703 | 0.965104519 | 5 | 2 | 0 | 0 | 19.81 |
| *Glyma.02g148200* | 0 | 0 | 25.806 | 0 | 4 | 15 | 36 | 62.81 | *Glyma.07g036400* | 9 | 6.684029655 | 2.015378786 | 0 | 2 | 0 | 0 | 19.70 |
| *Glyma.12g094100* | 0 | 0 | 25.806 | 0 | 4 | 15 | 36 | 62.81 | *Glyma.10g048000* | 9 | 7.051098239 | 1.25259 | 0 | 2 | 0 | 0 | 19.30 |
| *Glyma.13g243600* | 30 | 27 | 0.2056 | 0 | 4 | 0 | 0 | 61.21 | *Glyma.08g128100* | 6.493436351 | 5.35261703 | 4.661929423 | 0 | 2 | 0 | 0 | 18.51 |
| *Glyma.13g243900* | 30 | 27 | 0 | 0 | 4 | 0 | 0 | 61.00 | *Glyma.05g108900* | 0 | 4 | 6.451544993 | 6 | 2 | 0 | 0 | 18.45 |
| *Glyma.05g108900* | 0 | 12 | 25.806 | 18 | 4 | 0 | 0 | 59.81 | *Glyma.16g018500* | 0 | 4 | 6.451544993 | 6 | 2 | 0 | 0 | 18.45 |
| *Glyma.16g018500* | 0 | 12 | 25.806 | 18 | 4 | 0 | 0 | 59.81 | *Glyma.10g048100* | 9 | 7.051098239 | 0.274604052 | 0 | 2 | 0 | 0 | 18.33 |
| *Glyma.07g036400* | 27 | 20.05209 | 8.0615 | 0 | 4 | 0 | 0 | 59.11 | *Glyma.10g047800* | 9 | 7.051098239 | 0.1189775 | 0 | 2 | 0 | 0 | 18.17 |
| *Glyma.08g128500* | 19.48 | 16.05785 | 3.8604 | 15 | 4 | 0 | 0 | 58.40 | *Glyma.10g048200* | 9 | 7.051098239 | 0.081765 | 0 | 2 | 0 | 0 | 18.13 |
| *Glyma.08g128100* | 19.48 | 16.05785 | 18.648 | 0 | 4 | 0 | 0 | 58.19 | *Glyma.07g036300* | 9 | 6.684029655 | 0.403675384 | 0 | 2 | 0 | 0 | 18.09 |
| *Glyma.10g048000* | 27 | 21.15329 | 5.0104 | 0 | 4 | 0 | 0 | 57.16 | *Glyma.07g036200* | 9 | 6.684029655 | 0.161786 | 0 | 2 | 0 | 0 | 17.85 |
| *Glyma.09g153900* | 0 | 0 | 19.304 | 0 | 4 | 15 | 36 | 56.30 | *Glyma.07g036100* | 9 | 6.684029655 | 0.02043345 | 0 | 2 | 0 | 0 | 17.70 |
| *Glyma.16g204600* | 0 | 0 | 18.958 | 0 | 4 | 15 | 36 | 55.96 | *Glyma.10g047900* | 9 | 7.051098239 | 0.0212702 | 0 | 1 | 0 | 0 | 17.07 |
| *Glyma.12g150500* | 0 | 0 | 25.806 | 0 | 4 | 7.143 | 36 | 54.95 | *Glyma.14g202300* | 10 | 0 | 1.5 | 0 | 5 | 0 | 0 | 16.50 |
| *Glyma.13g208000* | 0 | 0 | 25.806 | 0 | 4 | 6.827 | 36 | 54.63 | *Glyma.14g121200* | 0 | 0 | 6.451544993 | 0 | 2 | 5 | 3 | 16.45 |
| *Glyma.08g199800* | 0 | 0 | 25.806 | 0 | 4 | 6.307 | 36 | 54.11 | *Glyma.03g132700* | 0 | 0 | 6.451544993 | 0 | 2 | 5 | 3 | 16.45 |
| *Glyma.12g222400* | 0 | 0 | 25.806 | 0 | 4 | 6.067 | 36 | 53.87 | *Glyma.11g179300* | 0 | 0 | 6.451544993 | 0 | 2 | 5 | 3 | 16.45 |
| *Glyma.10g048100* | 27 | 21.15329 | 1.0984 | 0 | 4 | 0 | 0 | 53.25 | *Glyma.02g148200* | 0 | 0 | 6.451544993 | 0 | 2 | 5 | 3 | 16.45 |
| *Glyma.07g036300* | 27 | 20.05209 | 1.6147 | 0 | 4 | 0 | 0 | 52.67 | *Glyma.12g094100* | 0 | 0 | 6.451544993 | 0 | 2 | 5 | 3 | 16.45 |
| *Glyma.10g047800* | 27 | 21.15329 | 0.4759 | 0 | 4 | 0 | 0 | 52.63 | *Glyma.04g044900* | 0 | 0 | 6.451544993 | 5 | 5 | 0 | 0 | 16.45 |
| *Glyma.10g048200* | 27 | 21.15329 | 0.3271 | 0 | 4 | 0 | 0 | 52.48 | *Glyma.08g218600* | 0 | 0 | 6.451544993 | 5 | 5 | 0 | 0 | 16.45 |
| *Glyma.05g123900* | 0 | 0 | 25.806 | 15 | 4 | 7.331 | 0 | 52.14 | *Glyma.05g123900* | 0 | 0 | 6.451544993 | 5 | 2 | 2.443697499 | 0 | 15.90 |
| *Glyma.01g118000* | 0 | 0 | 25.806 | 0 | 4 | 4.288 | 36 | 52.09 | *Glyma.08g139100* | 0 | 10 | 3.891873012 | 0 | 2 | 0 | 0 | 15.90 |
| *Glyma.07g036200* | 27 | 20.05209 | 0.6471 | 0 | 4 | 0 | 0 | 51.70 | *Glyma.08g119200* | 0 | 10 | 3.797615828 | 0 | 2 | 0 | 0 | 15.80 |
| *Glyma.05g124000* | 0 | 0 | 25.806 | 15 | 4 | 6.732 | 0 | 51.54 | *Glyma.10g180800* | 0 | 0 | 5.755530814 | 5 | 5 | 0 | 0 | 15.76 |
| *Glyma.07g036100* | 27 | 20.05209 | 0.0817 | 0 | 4 | 0 | 0 | 51.13 | *Glyma.05g124000* | 0 | 0 | 6.451544993 | 5 | 2 | 2.244125144 | 0 | 15.70 |
| *Glyma.04g044900* | 0 | 0 | 25.806 | 15 | 10 | 0 | 0 | 50.81 | *Glyma.08g119600* | 0 | 10 | 3.661767907 | 0 | 2 | 0 | 0 | 15.66 |
| *Glyma.08g218600* | 0 | 0 | 25.806 | 15 | 10 | 0 | 0 | 50.81 | *Glyma.08g119000* | 0 | 10 | 3.525234625 | 0 | 2 | 0 | 0 | 15.53 |
| *Glyma.13g250400* | 20.99 | 0 | 25.806 | 0 | 4 | 0 | 0 | 50.80 | *Glyma.13g279900* | 0 | 0 | 5.523232184 | 5 | 5 | 0 | 0 | 15.52 |
| *Glyma.07g032900* | 0 | 20.58036 | 25.806 | 0 | 4 | 0 | 0 | 50.39 | *Glyma.13g250400* | 6.998025567 | 0 | 6.451544993 | 0 | 2 | 0 | 0 | 15.45 |
| *Glyma.10g047900* | 27 | 21.15329 | 0.0851 | 0 | 2 | 0 | 0 | 50.24 | *Glyma.08g119100* | 0 | 10 | 3.410996354 | 0 | 2 | 0 | 0 | 15.41 |
| *Glyma.13g270100* | 0 | 0 | 20.905 | 0 | 4 | 7.227 | 36 | 50.13 | *Glyma.07g032900* | 0 | 6.860120914 | 6.451544993 | 0 | 2 | 0 | 0 | 15.31 |
| *Glyma.07g153100* | 0 | 0 | 19.561 | 0 | 4 | 8.472 | 36 | 50.03 | *Glyma.13g251100* | 9 | 0 | 4.26154654 | 0 | 2 | 0 | 0 | 15.26 |
| *Glyma.18g009700* | 0 | 0 | 21.952 | 0 | 4 | 5.741 | 36 | 49.70 | *Glyma.07g031400* | 0 | 10 | 3.17529991 | 0 | 2 | 0 | 0 | 15.25 |
| *Glyma.08g139100* | 0 | 30 | 15.567 | 0 | 4 | 0 | 0 | 49.57 | *Glyma.08g119500* | 0 | 0 | 4.825927696 | 0 | 2 | 5 | 3 | 15.18 |
| *Glyma.08g119200* | 0 | 30 | 15.19 | 0 | 4 | 0 | 0 | 49.19 | *Glyma.09g153900* | 0 | 10 | 3.82204579 | 0 | 1 | 0 | 0 | 14.86 |
| *Glyma.14g202300* | 30 | 0 | 9 | 0 | 10 | 0 | 0 | 49.00 | *Glyma.08g119400* | 0 | 0 | 4.739412478 | 0 | 2 | 5 | 3 | 14.82 |
| *Glyma.08g119600* | 0 | 30 | 14.647 | 0 | 4 | 0 | 0 | 48.65 | *Glyma.16g204600* | 0 | 0 | 6.451544993 | 6 | 2 | 0 | 0 | 14.74 |
| *Glyma.08g119000* | 0 | 30 | 14.101 | 0 | 4 | 0 | 0 | 48.10 | *Glyma.17g147500* | 0 | 0 | 6.451544993 | 6 | 2 | 0 | 0 | 14.67 |
| *Glyma.13g251100* | 27 | 0 | 17.046 | 0 | 4 | 0 | 0 | 48.05 | *Glyma.14g049000* | 0 | 0 | 6.451544993 | 6 | 2 | 0 | 0 | 14.45 |
| *Glyma.10g180800* | 0 | 0 | 23.022 | 15 | 10 | 0 | 0 | 48.02 | *Glyma.07g105700* | 0 | 0 | 6.451544993 | 6 | 2 | 0 | 0 | 14.45 |
| *Glyma.14g049000* | 0 | 0 | 25.806 | 18 | 4 | 0 | 0 | 47.81 | *Glyma.12g093100* | 0 | 0 | 6.451544993 | 6 | 2 | 0 | 0 | 14.45 |
| *Glyma.07g105700* | 0 | 0 | 25.806 | 18 | 4 | 0 | 0 | 47.81 | *Glyma.09g172500* | 0 | 0 | 6.451544993 | 6 | 2 | 0 | 0 | 14.45 |
| *Glyma.12g093100* | 0 | 0 | 25.806 | 18 | 4 | 0 | 0 | 47.81 | *Glyma.12g187400* | 0 | 0 | 6.451544993 | 6 | 2 | 0 | 0 | 14.45 |
| *Glyma.09g172500* | 0 | 0 | 25.806 | 18 | 4 | 0 | 0 | 47.81 | *Glyma.08g083300* | 0 | 0 | 6.451544993 | 6 | 2 | 0 | 0 | 14.45 |
| *Glyma.12g187400* | 0 | 0 | 25.806 | 18 | 4 | 0 | 0 | 47.81 | *Glyma.02g268200* | 0 | 0 | 6.451544993 | 6 | 2 | 0 | 0 | 14.45 |
| *Glyma.08g083300* | 0 | 0 | 25.806 | 18 | 4 | 0 | 0 | 47.81 | *Glyma.03g173300* | 0 | 0 | 6.451544993 | 6 | 2 | 0 | 0 | 14.45 |
| *Glyma.02g268200* | 0 | 0 | 25.806 | 18 | 4 | 0 | 0 | 47.81 | *Glyma.18g042100* | 0 | 0 | 6.451544993 | 6 | 2 | 0 | 0 | 14.45 |
| *Glyma.03g173300* | 0 | 0 | 25.806 | 18 | 4 | 0 | 0 | 47.81 | *Glyma.17g174500* | 0 | 0 | 6.451544993 | 6 | 2 | 0 | 0 | 14.45 |
| *Glyma.18g042100* | 0 | 0 | 25.806 | 18 | 4 | 0 | 0 | 47.81 | *Glyma.11g181200* | 0 | 0 | 6.451544993 | 6 | 2 | 0 | 0 | 14.45 |
| *Glyma.17g174500* | 0 | 0 | 25.806 | 18 | 4 | 0 | 0 | 47.81 | *Glyma.06g100900* | 0 | 0 | 6.451544993 | 6 | 2 | 0 | 0 | 14.45 |
| *Glyma.11g181200* | 0 | 0 | 25.806 | 18 | 4 | 0 | 0 | 47.81 | *Glyma.05g128200* | 0 | 0 | 6.451544993 | 6 | 2 | 0 | 0 | 14.45 |
| *Glyma.06g100900* | 0 | 0 | 25.806 | 18 | 4 | 0 | 0 | 47.81 | *Glyma.07g049900* | 0 | 0 | 6.451544993 | 6 | 2 | 0 | 0 | 14.45 |
| *Glyma.05g128200* | 0 | 0 | 25.806 | 18 | 4 | 0 | 0 | 47.81 | *Glyma.04g092100* | 0 | 0 | 6.451544993 | 6 | 2 | 0 | 0 | 14.45 |
| *Glyma.07g049900* | 0 | 0 | 25.806 | 18 | 4 | 0 | 0 | 47.81 | *Glyma.17g158100* | 0 | 0 | 6.451544993 | 6 | 2 | 0 | 0 | 14.45 |
| *Glyma.04g092100* | 0 | 0 | 25.806 | 18 | 4 | 0 | 0 | 47.81 | *Glyma.07g253700* | 0 | 0 | 6.451544993 | 6 | 2 | 0 | 0 | 14.45 |
| *Glyma.17g158100* | 0 | 0 | 25.806 | 18 | 4 | 0 | 0 | 47.81 | *Glyma.09g149200* | 0 | 0 | 6.379674782 | 6 | 2 | 0 | 0 | 14.45 |
| *Glyma.07g253700* | 0 | 0 | 25.806 | 18 | 4 | 0 | 0 | 47.81 | *Glyma.20g218100* | 9 | 0 | 4.301029996 | 0 | 1 | 0 | 0 | 14.45 |
| *Glyma.09g149200* | 0 | 0 | 25.806 | 18 | 4 | 0 | 0 | 47.81 | *Glyma.11g180500* | 6.998025567 | 0 | 5.284318118 | 0 | 2 | 0 | 0 | 14.38 |
| *Glyma.20g218100* | 0 | 0 | 25.806 | 18 | 4 | 0 | 0 | 47.81 | *Glyma.08g153000* | 0 | 0 | 6.216579725 | 6 | 2 | 0 | 0 | 14.33 |
| *Glyma.08g119100* | 0 | 30 | 13.644 | 0 | 4 | 0 | 0 | 47.64 | *Glyma.13g251300* | 0 | 0 | 6.213095891 | 6 | 2 | 0 | 0 | 14.30 |
| *Glyma.11g180500* | 0 | 0 | 25.519 | 18 | 4 | 0 | 0 | 47.52 | *Glyma.13g250300* | 0 | 0 | 6.116559272 | 6 | 2 | 0 | 0 | 14.28 |
| *Glyma.08g119400* | 0 | 30 | 15.288 | 0 | 2 | 0 | 0 | 47.29 | *Glyma.19g213300* | 0 | 0 | 6.089462783 | 6 | 2 | 0 | 0 | 14.22 |
| *Glyma.13g279900* | 0 | 0 | 22.093 | 15 | 10 | 0 | 0 | 47.10 | *Glyma.20g209700* | 0 | 0 | 6.032646177 | 6 | 2 | 0 | 0 | 14.21 |
| *Glyma.19g213300* | 0 | 0 | 24.866 | 18 | 4 | 0 | 0 | 46.87 | *Glyma.08g176300* | 0 | 0 | 6 | 6 | 2 | 0 | 0 | 14.21 |
| *Glyma.08g176300* | 0 | 0 | 24.852 | 18 | 4 | 0 | 0 | 46.85 | *Glyma.08g138600* | 0 | 0 | 5.961571656 | 6 | 2 | 0 | 0 | 14.20 |
| *Glyma.08g119500* | 0 | 30 | 12.701 | 0 | 4 | 0 | 0 | 46.70 | *Glyma.13g234500* | 0 | 0 | 6.451544993 | 0 | 2 | 2.380952381 | 3 | 14.12 |
| *Glyma.13g234500* | 0 | 0 | 24.466 | 18 | 4 | 0 | 0 | 46.47 | *Glyma.10g073600* | 0 | 0 | 6.451544993 | 0 | 2 | 2.27572413 | 3 | 14.09 |
| *Glyma.10g073600* | 0 | 0 | 24.358 | 18 | 4 | 0 | 0 | 46.36 | *Glyma.01g037200* | 0 | 0 | 6.451544993 | 0 | 2 | 2.102372909 | 3 | 14.03 |
| *Glyma.13g251300* | 27 | 0 | 17.204 | 0 | 2 | 0 | 0 | 46.20 | *Glyma.19g174200* | 0 | 0 | 6.451544993 | 0 | 2 | 2.022276395 | 3 | 14.00 |
| *Glyma.13g250300* | 20.99 | 0 | 21.137 | 0 | 4 | 0 | 0 | 46.13 | *Glyma.17g020600* | 0 | 0 | 6.451544993 | 6 | 1 | 0 | 0 | 13.96 |
| *Glyma.01g037200* | 0 | 0 | 24.131 | 18 | 4 | 0 | 0 | 46.13 | *Glyma.08g128700* | 0 | 0 | 6.451544993 | 0 | 2 | 1.42945706 | 3 | 13.95 |
| *Glyma.19g174200* | 0 | 0 | 24 | 18 | 4 | 0 | 0 | 46.00 | *Glyma.08g128300* | 0 | 0 | 4.89029261 | 0 | 2 | 2.823908741 | 3 | 13.92 |
| *Glyma.11g149900* | 0 | 0 | 17.563 | 0 | 4 | 6.392 | 36 | 45.95 | *Glyma.08g128200* | 0 | 0 | 5.226203053 | 0 | 2 | 2.408935393 | 3 | 13.85 |
| *Glyma.17g020600* | 0 | 0 | 23.846 | 18 | 4 | 0 | 0 | 45.85 | *Glyma.12g150500* | 0 | 0 | 5.488092769 | 0 | 2 | 1.913640169 | 3 | 13.83 |
| *Glyma.17g205000* | 0 | 0 | 25.806 | 18 | 2 | 0 | 0 | 45.81 | *Glyma.03g015800* | 0 | 0 | 4.390770987 | 0 | 2 | 2.13076828 | 3 | 13.74 |

**Supplementary Table** **7.** Gene mapping results between ±20 kb of 27 SSR molecular marker in previous studies.

| #SSR | #Chr | #Start | #End | #±20 kb Mapping genes (v2.0) | #Reference |
| --- | --- | --- | --- | --- | --- |
| Sat_064 | 18 | 56333703 | 56333845 | *Glyma.18g282300, Glyma.18g282400, Glyma.18g282500* | Cregan et al. (1999) |
| Sat_351 | 2 | 4904997 | 4905271 | \| *Glyma.02g053600,* \| \| --- \| \| *Glyma.02g053800,* \| \| *Glyma.02g053700,* \| \| *Glyma.02g053900,* \| \| *Glyma.02g054000,* \| \| *Glyma.02g054100,* \| \| *Glyma.02g054200,* \| \| *Glyma.02g054300* \| | Sayama et al. (2009) |
| Sat_279 | 2 | 2005085 | 2005345 | *Glyma.02g022300* | Sayama et al. (2009) |
| Satt180 | 4 | 47299563 | 47299610 | \| *Glyma.04g236000,* \| \| --- \| \| *Glyma.04g236100,* \| \| *Glyma.04g236200* \| | Sayama et al. (2009) |
| Satt338 | 4 | 50092364 | 50092608 | \| *Glyma.04g232500,* \| \| --- \| \| *Glyma.04g232600,* \| \| *Glyma.04g232700* \| | Sayama et al. (2009) |
| AW132402 | 8 | 11786768 | 11786918 | \| *Glyma.08g152900,* \| \| --- \| \| *Glyma.08g153000,* \| \| *Glyma.08g153100* \| | Sayama et al. (2009) |
| Satt424 | 8 | 10633645 | 10633908 | \| *Glyma.08g138600,* \| \| --- \| \| *Glyma.08g138700,* \| \| *Glyma.08g138800,* \| \| *Glyma.08g138900,* \| \| *Glyma.08g139000,* \| \| *Glyma.08g139100,* \| \| *Glyma.08g139200* \| | Sayama et al. (2009) |
| Satt187 | 8 | 9192408 | 9192679 | \| *Glyma.08g118700,* \| \| --- \| \| *Glyma.08g118800,* \| \| *Glyma.08g118900,* \| \| *Glyma.08g119000,* \| \| *Glyma.08g119100,* \| \| *Glyma.08g119200,* \| \| *Glyma.08g119300,* \| \| *Glyma.08g119400,* \| \| *Glyma.08g119500* \| | Sayama et al. (2009) |
| Sat_218 | 12 | 37556592 | 37556872 | \| *Glyma.12g216200,* \| \| --- \| \| *Glyma.12g216300,* \| \| *Glyma.12g216400,* \| \| *Glyma.12g216500* \| | Sayama et al. (2009) |
| Satt581 | 10 | 44713925 | 44714068 | \| *Glyma.10g214800,* \| \| --- \| \| *Glyma.10g214900,* \| \| *Glyma.10g215000,* \| \| *Glyma.10g215100* \| | Cregan et al. (1999) |
| Satt153 | 10 | 45959176 | 45959187 | \| *Glyma.10g236200,* \| \| --- \| \| *Glyma.10g236300,* \| \| *Glyma.10g236400,* \| \| *Glyma.10g236500* \| | Cregan et al. (1999) |
| Satt658 | 6 | 20754844 | 20755065 | \| *Glyma.06g210200,* \| \| --- \| \| *Glyma.06g210300* \| | Githiri et al. (2006) |
| Satt277 | 6 | 17218677 | 17218911 | *Glyma.06g193600* | Cregan et al. (1999) |
| Satt377 | 8 | 16361908 | 16362087 | \| *Glyma.08g201700,* \| \| --- \| \| *Glyma.08g201800,* \| \| *Glyma.08g201900* \| | Rizal and Karki (2011) |
| Satt499 | 9 | 37987699 | 37987773 | \| *Glyma.09g180800,* \| \| --- \| \| *Glyma.09g180900,* \|   *Glyma.09g181000* | Rizal and Karki (2011) |
| Sat_309 | 13 | 23683604 | 23683898 | \| *Glyma.13g123500,* \| \| --- \| \| *Glyma.13g123600,* \| \| *Glyma.13g123700,* \| | Rizal and Karki (2011) |
| Sat_134 | 19 | 14048834 | 14049112 | *Glyma.19g063600* | Rizal and Karki (2011) |
| Satt184 | 1 | 1473591 | 1473754 | \| *Glyma.01g014800,* \| \| --- \| \| *Glyma.01g014900,* \| \| *Glyma.01g015000* \| | Rizal and Karki (2011) |
| Satt296 | 2 | 13335767 | 13335951 | \| *Glyma.02g129800,* \| \| --- \| \| *Glyma.02g129900,* \| \| *Glyma.02g130000,* \| \| *Glyma.02g130100,* \| \| *Glyma.02g130200* \| | Githiri et al. (2006) |
| Satt164 | 4 | 47677317 | 47677338 | *Glyma.04g239500,*   \| *Glyma.04g239600,* \| \| --- \| \| *Glyma.04g239700* \| | Githiri et al. (2006) |
| Satt477 | 10 | 40306793 | 40306950 | \| *Glyma.10g168700,* \| \| --- \| \| *Glyma.10g168800,* \| \| *Glyma.10g168900* \| | Githiri et al. (2006) |
| Satt290 | 2 | 28177818 | 28177837 | *Glyma.05g107700* | Githiri et al. (2006) |
| Satt252 | 13 | 16454986 | 16455201 | \| *Glyma.13g064900,* \| \| --- \| \| *Glyma.13g065000,* \| \| *Glyma.13g065100,* \| \| *Glyma.13g065200,* \| \| *Glyma.13g065300* \| | Cornelious et al. (2005) |
| Satt385 | 5 | 35536817 | 35537127 | \| *Glyma.05g164300,* \| \| --- \| \| *Glyma.05g164400,* \| \| *Glyma.05g164500,* \| \| *Glyma.05g164600,* \| \| *Glyma.05g164700* \| | Cornelious et al. (2005) |

Abbreviation: kb, kilo base pair; Chr, chromosome.

**REFERENCES**

Cornelious, B., Chen, P., Chen, Y., de Leon, N., Shannon, J.G., and Wang, D. (2005). Identification of QTLs underlying water-logging tolerance in soybean. *Molecular Breeding* 16(2)**,** 103-112. doi: 10.1007/s11032-005-5911-2.

Cregan, P.B., Jarvik, T., Bush, A.L., Shoemaker, R.C., Lark, K.G., Kahler, A.L., et al. (1999). An integrated genetic linkage map of the soybean genome. *Crop Sci* 39. doi: 10.2135/cropsci1999.3951464x.

Githiri, S.M., Watanabe, S., Harada, K., and and Takahashi, R. (2006). QTL analysis of flooding tolerance in soybean at an early vegetative growth stage. *Plant Breeding* 125**,** 613-618.

Rizal, G., and Karki, S. (2011). Alcohol dehydrogenase (ADH) activity in soybean [*Glycine max* (L.) Merr.] under flooding stress. *Electronic Journal of Plant Breeding* 2(1)**,** 50-57.

Sayama, T., Nakazaki, T., Ishikawa, G., Yagasaki, K., Yamada, N., Hirota, N., et al. (2009). QTL analysis of seed-flooding tolerance in soybean [*Glycine max* (L.) Merr.]. *Plant Sci* 176(4)**,** 514-521. doi: 10.1016/j.plantsci.2009.01.007.

**Supplementary Table** **8.** Fifty-nine soybean flooding tolerance prioritized genes identified in Kim et al. (2017).

| *Glyma.01g095000* | *Glyma.09g153900* | *Glyma.15g073200* | *Glyma.U012100* |
| --- | --- | --- | --- |
| *Glyma.01g118000* | *Glyma.09g166000* | *Glyma.16g045000* | *Glyma.U032500* |
| *Glyma.02g068500* | *Glyma.11g149900* | *Glyma.16g045000* | *Glyma.03g223000* |
| *Glyma.02g148200* | *Glyma.11g179300* | *Glyma.16g150000* | *Glyma.15g190500* |
| *Glyma.02g222400* | *Glyma.11g245600* | *Glyma.16g200600* | *Glyma.19g220200* |
| *Glyma.03g055100* | *Glyma.11g255000* | *Glyma.16g204600* |  |
| *Glyma.03g132700* | *Glyma.12g094100* | *Glyma.16g216100* |  |
| *Glyma.03g244800* | *Glyma.12g095700* | *Glyma.17g011500* |  |
| *Glyma.04g086900* | *Glyma.12g150500* | *Glyma.17g138500* |  |
| *Glyma.04g213900* | *Glyma.12g190100* | *Glyma.17g164100* |  |
| *Glyma.05g103200* | *Glyma.12g222400* | *Glyma.18g009700* |  |
| *Glyma.05g124000* | *Glyma.13g208000* | *Glyma.18g011600* |  |
| *Glyma.05g224900* | *Glyma.13g231700* | *Glyma.19g017200* |  |
| *Glyma.06g088600* | *Glyma.13g240100* | *Glyma.19g106700* |  |
| *Glyma.07g014300* | *Glyma.13g261400* | *Glyma.19g242300* |  |
| *Glyma.07g153100* | *Glyma.13g270100* | *Glyma.20g025500* |  |
| *Glyma.08g078900* | *Glyma.13g311600* | *Glyma.20g072400* |  |
| *Glyma.08g199800* | *Glyma.14g121200* | *Glyma.20g189300* |  |

**Supplementary Table** **9.** The 117 genes mapped from GWAS data. Detailed information can be found in Yu et al. (2019).

| *Glyma.01g003000* | *Glyma.08g121500* | *Glyma.08g177800* | *Glyma.13g249900* |
| --- | --- | --- | --- |
| *Glyma.01g003100* | *Glyma.08g121600* | *Glyma.08g177900* | *Glyma.13g250000* |
| *Glyma.01g003200* | *Glyma.08g121700* | *Glyma.08g178000* | *Glyma.13g250100* |
| *Glyma.01g003300* | *Glyma.08g121800* | *Glyma.10g047800* | *Glyma.13g250200* |
| *Glyma.01g003400* | *Glyma.08g121900* | *Glyma.10g047900* | *Glyma.13g250300* |
| *Glyma.01g003500* | *Glyma.08g126300* | *Glyma.10g048000* | *Glyma.13g250400* |
| *Glyma.01g003600* | *Glyma.08g126400* | *Glyma.10g048100* | *Glyma.13g250700* |
| *Glyma.01g003700* | *Glyma.08g126500* | *Glyma.10g048200* | *Glyma.13g250800* |
| *Glyma.01g003800* | *Glyma.08g126600* | *Glyma.11g055300* | *Glyma.13g250900* |
| *Glyma.02g057600* | *Glyma.08g126700* | *Glyma.11g055400* | *Glyma.13g251000* |
| *Glyma.02g057700* | *Glyma.08g126800* | *Glyma.11g055500* | *Glyma.13g251100* |
| *Glyma.02g057800* | *Glyma.08g126900* | *Glyma.11g055600* | *Glyma.13g251200* |
| *Glyma.02g057900* | *Glyma.08g128100* | *Glyma.11g055700* | *Glyma.13g251300* |
| *Glyma.02g058000* | *Glyma.08g128200* | *Glyma.13g237900* | *Glyma.13g259000* |
| *Glyma.07g031100* | *Glyma.08g128300* | *Glyma.13g238000* | *Glyma.13g259100* |
| *Glyma.07g031200* | *Glyma.08g128400* | *Glyma.13g238100* | *Glyma.13g259200* |
| *Glyma.07g031300* | *Glyma.08g128500* | *Glyma.13g238200* | *Glyma.13g259300* |
| *Glyma.07g031400* | *Glyma.08g128600* | *Glyma.13g243600* | *Glyma.14g202100* |
| *Glyma.07g031500* | *Glyma.08g128700* | *Glyma.13g243700* | *Glyma.14g202200* |
| *Glyma.07g031600* | *Glyma.08g135800* | *Glyma.13g243800* | *Glyma.14g202300* |
| *Glyma.07g036100* | *Glyma.08g135900* | *Glyma.13g243900* | *Glyma.14g202400* |
| *Glyma.07g036200* | *Glyma.08g136000* | *Glyma.13g244000* | *Glyma.14g202500* |
| *Glyma.07g036300* | *Glyma.08g136100* | *Glyma.13g244100* | *Glyma.14g202600* |
| *Glyma.07g036400* | *Glyma.08g136200* | *Glyma.13g248100* | *Glyma.14g202700* |
| *Glyma.08g121000* | *Glyma.08g136300* | *Glyma.13g248200* | *Glyma.14g202800* |
| *Glyma.08g121100* | *Glyma.08g136400* | *Glyma.13g248300* | *Glyma.14g202900* |
| *Glyma.08g121200* | *Glyma.08g177500* | *Glyma.13g248400* | *Glyma.19g105700* |
| *Glyma.08g121300* | *Glyma.08g177600* | *Glyma.13g248500* |  |
| *Glyma.08g121400* | *Glyma.08g177700* | *Glyma.13g248600* |  |
| *Glyma.20g185700* | *Glyma.20g185600* | *Glyma.20g185500* |  |

**Supplementary Table** **10.** The list of 74 overlapped FTgenes between 83 weighted

genes and 83 unweighted genes.

| *Glyma.13g243800* | *Glyma.13g250400* | *Glyma.03g132700* | *Glyma.19g213300* |
| --- | --- | --- | --- |
| *Glyma.13g244000* | *Glyma.08g119100* | *Glyma.11g179300* | *Glyma.08g176300* |
| *Glyma.13g244100* | *Glyma.07g032900* | *Glyma.02g148200* | *Glyma.13g234500* |
| *Glyma.13g243700* | *Glyma.13g251100* | *Glyma.12g094100* | *Glyma.10g073600* |
| *Glyma.13g243600* | *Glyma.08g119500* | *Glyma.04g044900* | *Glyma.01g037200* |
| *Glyma.13g243900* | *Glyma.09g153900* | *Glyma.08g218600* | *Glyma.19g174200* |
| *Glyma.11g055700* | *Glyma.08g119400* | *Glyma.05g123900* | *Glyma.17g020600* |
| *Glyma.08g128500* | *Glyma.16g204600* | *Glyma.08g139100* | *Glyma.12g150500* |
| *Glyma.07g036400* | *Glyma.14g049000* | *Glyma.08g119200* |  |
| *Glyma.10g048000* | *Glyma.07g105700* | *Glyma.10g180800* |  |
| *Glyma.08g128100* | *Glyma.12g093100* | *Glyma.05g124000* |  |
| *Glyma.05g108900* | *Glyma.09g172500* | *Glyma.08g119600* |  |
| *Glyma.16g018500* | *Glyma.12g187400* | *Glyma.08g119000* |  |
| *Glyma.10g048100* | *Glyma.08g083300* | *Glyma.13g279900* |  |
| *Glyma.10g047800* | *Glyma.02g268200* | *Glyma.04g092100* |  |
| *Glyma.10g048200* | *Glyma.03g173300* | *Glyma.17g158100* |  |
| *Glyma.07g036300* | *Glyma.18g042100* | *Glyma.07g253700* |  |
| *Glyma.07g036200* | *Glyma.17g174500* | *Glyma.09g149200* |  |
| *Glyma.07g036100* | *Glyma.11g181200* | *Glyma.20g218100* |  |
| *Glyma.10g047900* | *Glyma.06g100900* | *Glyma.11g180500* |  |
| *Glyma.14g202300* | *Glyma.05g128200* | *Glyma.13g251300* |  |
| *Glyma.14g121200* | *Glyma.07g049900* | *Glyma.13g250300* |  |


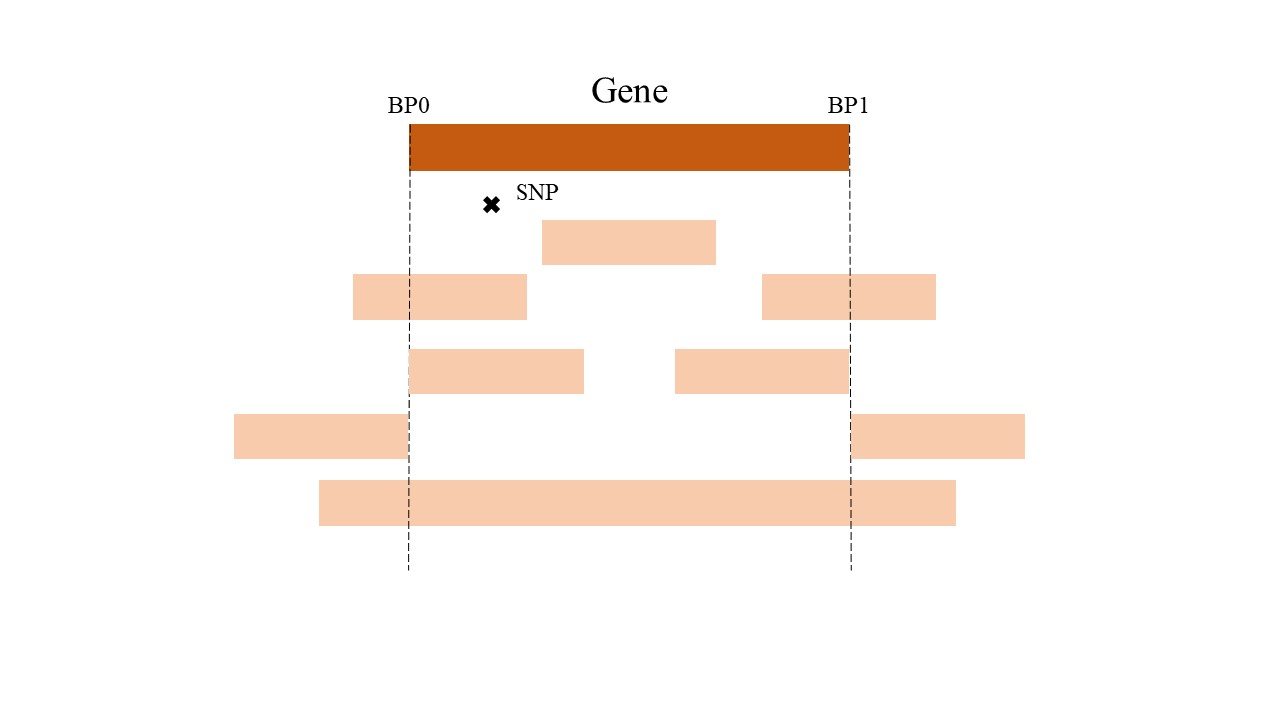


**Supplementary Figure** **1.** The principle of gene mapping. BP0 represents the start point of the gene sequence. BP1 represents the end point of the gene sequence. Red bands represent genotype data (SNP, SSR, etc.), and orange band represents the target gene.

**Supplementary Figure** **2.** Distribution of the quantity of 83 FTgenes on each soybean chromosome.
